# Advancing titanium dioxide coated photocatalytic depolluting surfaces: Leveraging ASINA's roadmap for safer and sustainable solutions

**DOI:** 10.1016/j.csbj.2024.10.001

**Published:** 2024-10-17

**Authors:** Irini Furxhi, Massimo Perucca, Giovanni Baldi, Valentina Dami, Andrea Cioni, Antti Joonas Koivisto, Rossella Bengalli, Paride Mantecca, Giulia Motta, Marie Carriere, Alessia Nicosia, Fabrizio Ravegnani, David Burrueco-Subirà, Socorro Vázquez-Campos, Elma Lahive, Jesús Lopez de Ipiña, Juliana Oliveira, Patrick Cronin, Magda Blosi, Anna Costa

**Affiliations:** a CNR-ISSMC Istituto di Scienza e Tecnologia dei Materiali Ceramici, Via Granarolo, 64, Faenza, RA 48018, Italy; b Project HUB360C.so Laghi 22, 10051 Avigliana, Turin, Italy; c COLOROBBIA Consulting srl, Via Pietramarina 53, Sovigliana-Vinci, FI 50059, Italy; d APM Air Pollution Management, Mattilanmäki 38, Tampere FI-33610, Finland; e INAR Institute for Atmospheric and Earth System Research, University of Helsinki, PL 64, FI-00014 UHEL, Helsinki, Finland; f ARCHE Consulting, Liefkensstraat 35D, Wondelgem B-9032, Belgium; g POLARIS Research Center, Dept. of Earth and Environmental Sciences, University of Milano Bicocca, Piazza della Scienza 1, Milano 20126, Italy; h CEA Univ. Grenoble Alpes, CNRS, Grenoble INP, IRIG, SYMMES, Grenoble 38000, France; i CNR-ISAC Institute of Atmospheric Sciences and Climate, Via Gobetti 101, Bologna 40129, Italy; j LEITAT Technological Center, C/ de la Innovació 2, Terrassa, Barcelona 08225, Spain; k Centre for Ecology & Hydrology (UKCEH), England, UK; l TECNALIA Research and Innovation - Basque Research and Technology Alliance (BRTA), Parque Tecnológico de Alava, Leonardo Da Vinci 11, Miñano 01510, Spain; m CeNTI - Centre of Nanotechnology and Smart Materials, Rua Fernando Mesquita 2785, Vila Nova de Famalicão, 4760–034, Portugal; n MicaNanotech Ltd, Limerick, Ireland

**Keywords:** Safe and sustainable by design, Titanium dioxide nanomaterials, Spray coating, Life-cycle, Roadmap

## Abstract

This report, the second of its kind from ASINA project, aims at providing a roadmap with quantitative metrics for Safe(r) and (more) Sustainable by Design (SSbD) solutions for titanium dioxide (TiO_2_) nanomaterials (NMs). We begin with a brief description of ASINA’s methodology across the product lifecycle, highlighting the quantitative elements, such as the Key Performance Indicators (KPIs). We then propose a decision support tool for implementing SSbD objectives across various dimensions—functionality, cost, environment, and human health safety. This is followed by the main innovative findings, a consolidation of the technical processes involved, design rationales, experimental procedures, tools and models, used and developed, to deliver photocatalytic depolluting surfaces by spray- finishing techniques based on TiO_2_ NMs formulations. The roadmap is thoroughly described to inform similar projects through the integration of KPIs into SSbD methodologies, fostering data-driven decision-making. While specific results are beyond this report's scope, its primary aim is to demonstrate the roadmap (SSbD know-how) and promote SSbD-oriented innovation in nanotechnology. Finally, we provide a comparison of the approaches followed in two case studies that target different industrial sectors. This case-specific SSbD assessments provide a concrete exemplification of the addressed methodology that contributes to the efforts towards attaining a common roadmap for implementing SSbD solutions aligned with the EU’s Green Deal objectives.

## Graphical Abstract

**Figure:**
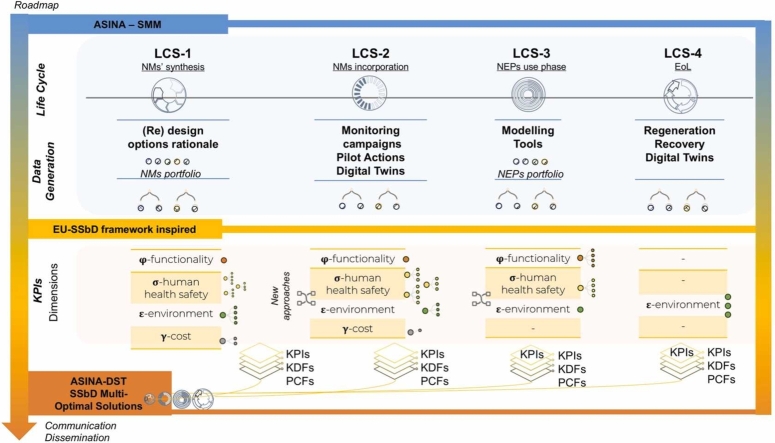

## 1 Introduction

The European (EU) Green Deal aims to transform the EU economy fostering a more sustainable future by implementing the United Nations' Agenda 2030 [1]. One of the key objectives of the agenda is the Zero Pollution Ambition. To achieve this, the Chemicals Strategy for Sustainability (CSS) was introduced in 2020, detailing a series of actions that promote safe and sustainable chemical practices [2]. In 2022, a recommendation known as the Safe and Sustainable by Desing (SSbD) framework, was published by the Commissions’ Joint Research Centre (JRC) [3]. In the evolving landscape of nanotechnology, the SSbD concept has emerged as a pivotal framework, guiding researchers, innovators, industry and policymakers towards the creation of advanced (nano)-materials (NMs),^1^ (nano-enabled) products (NEPs^2^) or processes that not only push the boundaries of innovation but also prioritize human and environmental health safety and sustainability along all the steps of the NMs/NEPs lifecycle. Such a concept is being intensively explored in the research field of nanotechnology [4], [5], [6]. Despite being a relatively new concept in the nanotechnology domain, SSbD has become an important component of many EU nanosafety projects^3^ [7], [8], [9], [10], [11]. In 2024, a methodological guidance was published by the JRC to clarify certain aspects of applying the SSbD framework, noting that it is a voluntary approach for professionals aiming to apply the framework in research and innovation and that it integrates multiple disciplines, primarily risk assessment and sustainability assessment, which vary in their established methodologies, framing, and ontologies [12]. Due to the variation of approaches and framings, stakeholders have identified that the primary challenge in implementing the SSbD framework is the difficulty in executing the assessment steps throughout the innovation process. As the field continues to mature (the JRC SSbD framework is expected to be updated and reissued in 2025), the quest for comprehensive quantitative metrics, approaches and methodologies that encapsulate the SSbD ethos are ongoing [12]. This report seeks to contribute to this dynamic discourse, by elucidating the conceptual underpinnings of the ASINA SSbD approach, and charting a course for future research and application. A detailed description of the i) ASINA SSbD Management Methodology (ASINA-SMM) and the projects objectives, the ii) three quantitative elements, defined from the data, models and tools and generated and used to guide decision-making: the Key Decision Factors (KDFs), the Key Performance Indicators (KPIs) and the Physical-Chemical Features (PCFs) associated to NMs, NEPs and processes and iii) the proposed ASINA decision support tool (ASINA-DST) can be found here [13].

## 2 Impact - results and outcomes

**Introduction to the Case Study:** The case study focuses on the synthesis and incorporation via spray-coating (spray-finishing when referred to textiles) techniques of ethanolic suspensions of TiO_2_ – N NMs into various substrates to produce photocatalytic coating active under visible light. Photocatalytic oxidation is a well-established method for degrading air pollutants into non-toxic or less harmful substances and deactivating viruses and bacteria [14]. Despite the acknowledged potential of visible light-activated TiO_2_ NMs in air purification technologies, their safety profiles in occupational and use scenarios continue to attract considerable attention. Classified as possibly carcinogenic to humans (Group 2B), the inhalable dose poses a potential risk. Additionally, concerns about its genotoxicity when ingested have led to its ban in the food sector [15], [16]. Alternative approaches are being explored in various sectors, such as medicinal products, to replace TiO_2_ or ensure the absence of nanosized TiO_2_ where necessary [17]. Given these considerations, adopting a SSbD approach is crucial, not to replace TiO_2_, but to innovate and design it in a way that mitigates potential human and environmental risks throughout the NMs lifecycle. This approach ensures safety and sustainability from the earliest stages of innovation, going beyond regulatory compliance to optimize TiO_2_ application in a responsible manner. In the following chapters, we provide a high-level overview of the technical aspects involved in defining the quantitative elements (KPIs, KDFs, and PCFs) including design rationales, experimental procedures, and tools/models used and developed across the entire NMs lifecycle, addressing all dimensions.

### 2.1 LCS-1: synthesis (production) phase

**(Re) design rationale:** TiO_2_ in its anatase form, is extensively studied for its photocatalytic activity, high specific surface area, and photochemical stability [18], [19]. However, due to its high band gap limits, its photocatalytic application is primarily in the ultraviolet (UV) range, which constitutes only 4 % of solar energy compared to 45 % in the visible light range [20]. Therefore, reducing the band gap to enhance absorption in the visible light region is necessary. This is achieved by doping TiO_2_ with metallic or non-metallic elements and coupling with other materials, shifting the required excitation energy closer to visible lights wavelengths [21]. The studied TiO_2_-based NMs portfolio was categorized into three levels to facilitate the SSbD assessment comparison and implementation process:

**-Baseline:** includes benchmarking NMs characterized in other EU nano-safety projects, such as TiO_2_ Sigma-Aldrich (Aeroxide® P25-JRC NM105), produced by high-temperature flame hydrolysis (aerosol process, Evonik). - **Tier 1:** includes engineered NMs with TRL 4–6, demonstrating scalability to lab-scale production and include: i) Nitrogen-doped TiO_2_ NMs (TiO_2_-N) patented by industrial partners Colorobbia through wet chemical preparation are designed to extend the applicability of TiO_2_ in indoor environments where sunlight or UV radiation is limited or absent. Unlike TiO_2_, which requires UV light for activation, TiO_2_-N can be activated by visible light (same light available indoors from artificial sources). ii) TiO_2_-@SiO_2_ composite NMs, heterocoagulated nanosolution in water suspension developed by ISSMC-CNR, containing 1.5 % wt TiO_2_ (Colorobbia) and 4.5 % wt SiO_2_. The SiO_2_ addition reduces the electron-hole recombination rate, extending radical availability and enhancing photocatalytic efficiency. SiO_2_ also improves particle size distribution homogeneity, stability, increased surface area and consequently enhanced activity against organic dyes in water [22] and Nitrogen Oxides (NOx) in air [23]. -**Tier 2:** includes variants of Tier 1 NMs further modified, such as multicomponent TiO_2_-N-@SiO_2_, developed by ISSMC-CNR through a colloidal process involving the heterocoagulation of dispersed phases.

**Fig. 1:**
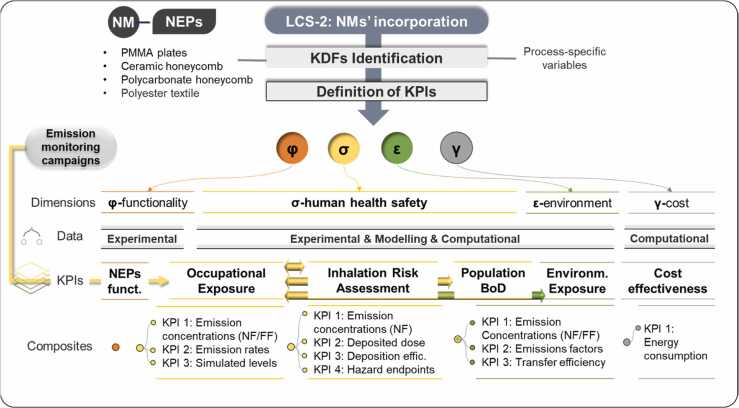
Overview of the (re)design strategy in LCS-1. Four dimensions are involved: φ-functionality, ε-environment, γ-cost and σ-safety (intrinsic).

The NMs portfolio (representing the KDF of material options) was synthesized and underwent physico-chemical (pchem) characterizations to ensure batch-to-batch reproducibility, stability over time and under various storage and handling conditions, providing the PCFs of the target NMs. In this LCS, we propose KPIs focusing on NMs’ functionality, environmental sustainability of the synthesis process, safety of NMs and cost-effectiveness (Fig. 1).

#### 2.1.1 φ-functionality dimension

The acceptance of NMs intended for industrial applications and commercialization must consider their intended functionality levels at the earliest stages of innovation.

**-NMs Functionality:**Table S1 (Supplementary material) presents a detailed outline of the KPI, including measurement methods, units, thresholds, objectives, and the associated KDFs and PCFs. This KPI assesses the photocatalytic efficiency of the TiO_2_ NMs portfolio involving laboratory testing under specific irradiation sources to degrade organic pollutants like Rhodamine B (RhB) dye. The reduction of the pollutant compound is quantified by UV-Vis absorption analysis and calculated as a percentage with the goal to maximize this KPI. KDFs include surface modifiers’ selection (uncoated/non-doped, N-doped, SiO₂-coated N-doped and SiO₂-coated), suspension concentration, irradiation duration, and irradiation source (visible or UV light). PCFs for functionality correlation purposes involve the primary particle size, hydrodynamic diameter (z-average) and polydispersity index (PdI), ζ-potential, specific surface area, band gap, crystalline phases, and turnover frequency (TOF). Additional PCFs included atomistic concentrations and crystallographic structures. These PCFs are indicative and may vary depending on the specific requirements and applications of the NMs.

#### 2.1.2 ε-environment dimension

Although the primary focus of the project was on Safe-bD approaches, sustainability elements were integrated due to rapid research advancements.

**-NMs synthesis sustainability:** In Table S2 is shown a composite KPI that assesses synthesis process contributions to global warming through greenhouse gas emissions. The functional unit is the amount of electricity required for the synthesis of 5 g of TiO_2_ NMs. A detailed description of the KPI can be found here [13]. The KPIs are not rigid in their selection. For example, a study proposed a framework based on a prospective LCA for early safety and sustainability assessment [11]. Environmental sustainability aspects, such as global warming potential and cumulative energy demand, along with toxicity aspects like human toxicity potential and freshwater ecotoxicity potential, were used. LCA is an evolving field of research, and there are currently no standardized sets of KPIs deemed more appropriate than others. The selection of KPIs in LCA studies vary depending on the specific goals of the assessment, the context of the study, the available data and methodologies.

#### 2.1.3 σ-human health safety dimension

This dimension assesses the potential human hazards of NMs based on their potential release during the lifecycle, which could lead to exposure for workers and end-users. For simplicity, we consider NMs as they are synthesised, acknowledging that they may transform during processing and end-of-life stages. A scoping analysis was conducted internally to identify the system boundaries for the hazard assessment within the NMs lifecycle, where (re)design efforts might have the greatest impact. The different KPIs explored in this assessment are illustrated in Fig. 1. A tiered approach based on Adverse Outcome Pathways (AOPs) outlined in Table S3, was implemented, and the following KPIs were evaluated, by using in vitro and acellular experimental models. The in vitro models used were human cell lines representative of NM target-organs, such as pulmonary (alveolar lung cells, A549), intestinal (HCT-116), and skin model (HaCat) to simulate effects at a cellular level of biological representation and at different routes of exposure to NMs (inhalation, ingestion and skin contact).

**- Intrinsic Inhalation Hazard**: The composite KPI (a detailed description of the KPIs can be found here [13] and in Table S4) contains i) cell viability (%), ii) oxidative stress (fold-change); iii) DNA damage (fold-change); iv) inflammatory potential (pg/mL or fold change) and v) cell-NMs bio-interaction using side scatter (SSC) values (au or fold change) obtained by cytofluorimetric analyses. **Intrinsic Ingestion Hazard**: Ingestion is another important route of NMs exposure to both workers and end-users [24]. Similar toxicological endpoints were utilised for this composite KPI (Table S4) and for the **Intrinsic Skin Hazard**. **Intrinsic Acellular Hazard:** The hazard potential of the TiO_2_ NMs portfolio was assessed by exploring their capacity to produce radicals and consume antioxidants using also acellular methods (Table S4). This evaluation focused on the ability of NMs to generate ROS, such as hydroxyl radicals (•OH). The assessment involved incubating the NMs with *N, N*-dimethyl-4-nitrosoaniline (RNO) and exposing them to UV irradiation to stimulate radical generation. Additionally, the consumption of antioxidants in an acellular environment was measured by evaluating the depletion of glutathione (GSH) and cysteine (Cys), both of which are crucial for cellular defence against oxidative stress. Two specific testing scenarios were considered: i) dark conditions - This scenario mimics environments devoid of light, evaluating the stability and reactivity of NMs without photoactivation. The combination of GSH, Cys, RNO, and Rhodamine B (RhB) models provides insight into whether nano-photocatalysts degrade RhB through direct oxidation or via •OH radical production; ii) UV irradiation conditions - this scenario assesses NM behaviour under light exposure, relevant for environments where NMs may be photoactivated.

#### 2.1.4 γ-cost dimension

Life Cycle Costing (LCC), as defined by EN ISO15686–5,^4^ is an economic assessment of relevant projected cost flows over a period of analysis, expressed in monetary terms.

-**NMs synthesis cost-effectiveness:** captures synthesis cost changes as a function of KDFs considering energy consumption and raw materials costs (total expenditure on final reagents used in the synthesis). For Tier 1 NMs, the cost for synthesizing the technical unit associated with 5 g of TiO_2_-N NMs was determined, and major cost contributors were analyzed. For Tier 2 NMs, the economic requirements were determined for the preparation of 1 kg of composite TiO_2_-@SiO_2_ NMs, focusing on the quantities of TiO_2_ and SiO_2_ used (Table S5).

### 2.2 LCS-2: NMs incorporation (processing) phase

NM suspensions were applied using spray coating techniques on four different substrates by depositing fine aerosol dispersions onto flexible or rigid surfaces:

− Hard plastic plates of polymethylmethacrylate (PMMA) pre-treated with plasma to maximise the formulation adhesion,
− Ceramic honeycomb;
− Polycarbonate honeycomb photocatalytic air filters for air purifiers. to be used as air filtration modules in air purifier systems, and
− Polyester (PES) textiles intended for technical fabrics such as UV-filtering and depollution-capable curtains.

**Fig. 2:**
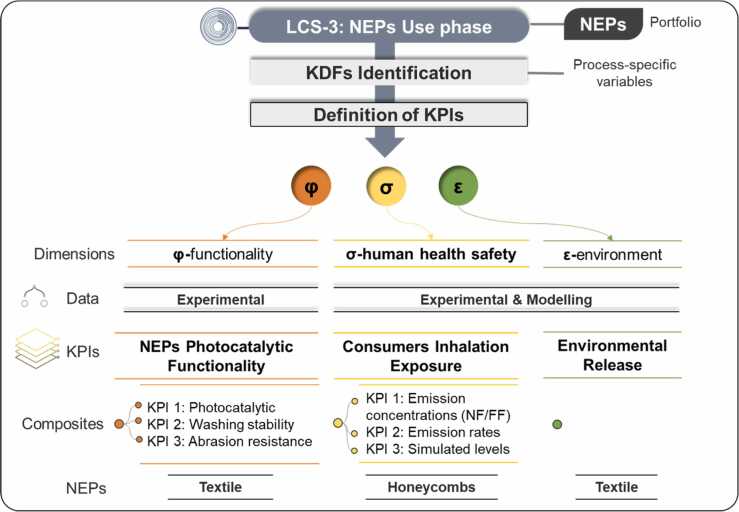
Summary of KPIs in the incorporation phase – human health and environmental related aspects, cost and functional KPIs are captured. Transversal to the KPIs, are KDFs representing specific process parameters.

The manufacturing stage involves depositing photocatalytic TiO₂ based NMs on PES textiles, PMMA plates, ceramic and polycarbonate honeycombs using primarily a spray finishing equipment at WITEK srl, Florence, Italy, including a pre-treatment plasma unit, a spray coating line, and a thermal drying station, all connected by a conveyor belt. This process is used to generate substrates for depolluting air filters or curtains as the final NEPs [25], enabling the deposition of NMs on substrates, with a capacity of up to 50 m² of coated substrate per day and a maximum width of 1.20 m, using a continuous roll-to-roll sequential process. Various KDFs such as the formulation’s sprayed onto the substrate and coating process parameters, were investigated. These KDFs are detailed in Table S6 (a unique set of KDFs tailored to the process). The incorporation phase involved several KPIs, as illustrated in Fig. 2.

#### 2.2.1 φ-functionality dimension

The functionality of NEPs is paramount in product development. Ensuring that NEPs perform their intended functions is crucial for meeting consumer expectations and maintaining marketability.

**- Functionality**: The indicator of functionality at LCS-2 is the minimum amount of NM deposited on NEPs that provides the desired level of photocatalytic activity. The latter is reported in LCS-3 (Section 2.5). In this case, the efficiency of the incorporation process is evaluated by measuring the amount of effective coating material applied, with the aim of minimizing sprayed material waste. The amount of NM deposited on PES and PMMA is evaluated through elemental analysis using inductively coupled plasma - optical emission spectrometry (ICP-OES) (Table S6). The KDFs, for this and all KPIs within this LCS, pertain to process-related factors, ensuring that each parameter is optimized to achieve the desired KPI.

##### 2.2.1.1 ASINA Pilot Action - MICA

As part of the ASINA’s pilot action for the manufacturing stage (LCS-2), a study focused on the functionality of NEP production process was conducted at a spray coating pilot plant (MICA, NanoTech, Ireland), aiming to optimize a simple and practical methodology for evaluating the quality of NEPs and the amount of deposited NMs. The subsequent KPIs considered were i) Quality: coating uniformity, demonstrated by bromophenol blue test; ii) Minimum amount of deposited material (grams per area of substrate), confirmed by particle distribution and ICP-OES to ensure materials’ efficiency; iii) Photocatalytic properties based on TiO_2_-coatings' emission in the UV region of the light spectrum. Textile treatment was achieved using a modification of the Textilise™ process from MICA. This process eliminates the use of binders and solvents, becoming more cost-effective and environmentally friendly. The fabrics were thermally activated before applying the agent, resulting in a more pronounced anchoring of the agent within the textile matrix. The KDFs considered included: textile composition (polyester/cotton 50/50 and 65/35 Polyester), composition and concentration of NM suspension, spray coating process duration and nozzle flow rate, conveyor belt speed (R2R) (60–3 m/min and 90–4,5 m/min) and thermal treatment temperature (between 80–84 °C). The proposed methodology offered an approach for obtaining a semi-quantitative estimation of the optimal spray coating process conditions for the production of NEP-coated textiles.

#### 2.2.2 σ-human health safety dimension

**-Occupational Exposure KPI:** Ensuring occupational safety and compliance with regulatory standards, is crucial for workers' health. Koivisto, Spinazzè [26] presented a tiered approach based on emission monitoring campaigns [25], [27] and probabilistic modelling to establish Conditions of Use (CoU) for exposure scenarios. Detailed documentation of this KPI can be found in a previous publication, which focused on a different case study [13] and in Table S7. The same KPI, along with identical approaches and tools, have been applied to the current case study. This ensures consistency and comparability across different case studies within the ASINA project.

**-Inhalation risk assessment**: Motta, Gualtieri [28] proposed a New Approach Methodology (NAM) in inhalation toxicology to assess potential hazards associated with the incorporation of pure TiO_2_ (NM-105) or TiO_2_-N (provided by Colorobbia, and freeze-dried by ISSMC-CNR) NMs. A detailed description of the KPIs can be found here [13] and in Table S7.

Beyond occupational safety, attention was also directed towards safeguarding the general population from potential inhalation exposure to TiO_2_-N NMs. The Burden of Disease (BoD) concept quantifies health impacts in Disability Adjusted Life Years (DALYs), an absolute metric that enables comparisons of health impacts from different causes. The BoD assessment is globally accepted and used by organizations such as the World Health Organization (WHO) and the European Environmental Agency. DALY is a time-based measure combining years of life lost due to premature mortality, years lived in states of less than full health, and years of healthy life lost due to disability. One DALY equals the loss of one healthy life year, and the burden of disease is the sum of these DALYs across the population. Applying the BoD concept to estimate health impacts from industrial NM emissions, resulted in a novel approach that provided a comprehensive assessment of the potential public health impacts from TiO_2_-N exposure. **Population Inhalation Exposure-Burden of Disease:** A multi-tier air emission assessment was developed based on monitoring campaigns and a bi-Gaussian plume model, IMPACT (Immission Prognosis Air Concentration Tool), to calculate the impact of industrial, residential, traffic, and agricultural emissions on local air concentrations and depositions on a 2 × 2 km grid [29], [30]. Based on air emissions data, exposure response functions for TiO_2_-N were used to calculate the BoD for the RWC production scenario (Table S7).

##### 2.2.2.1 ASINA pilot action -CeNTI

The validation step of SSbD strategies in real environment is crucial for their successful implementation. In ASINA, a pilot action established for the manufacturing stage (LCS-2). This involved a spray coating test bed at CeNTI (Portugal), focusing on NEP production, exposure assessment, and digital twin (DT) implementation [31] to validate the methodology hypothesis in a near-industrial environment (Table S6).

#### 2.2.3 ε-environment dimension

**-Environmental exposure:** Koivisto, Del Secco [29] established a quantitative foundation based on a tiered approach that incorporates data from monitoring campaigns (WITEK) to assess (i) spray coatings' environmental emissions and (ii) the environmental impacts of TiO_2_-N NMs (a detailed description of the KPIs can be found here [13] and in Table S8).

#### 2.2.4 γ-cost dimension

Keeping production and operational costs low while maintaining functionality is essential for competitive pricing. Efficient use of materials and energy not only reduces waste and operational costs but also contributes to overall sustainability. **NMs Incorporation Cost Effectiveness**: The LCC method, specific to the economic evaluation in the pilot action at CeNTI’s spray coating plant, according to ISO 15686, was used (a detailed description of the KPIs can be found here [13] and in Table S9). Representative costs include reagents, energy consumption, and infrastructure use (different conveyor belt speeds) per NEP unit quantity (cost required to treat 1 square meter of fabric).

### 2.3 LCS-3: NEPs use phase

**Fig. 3:**
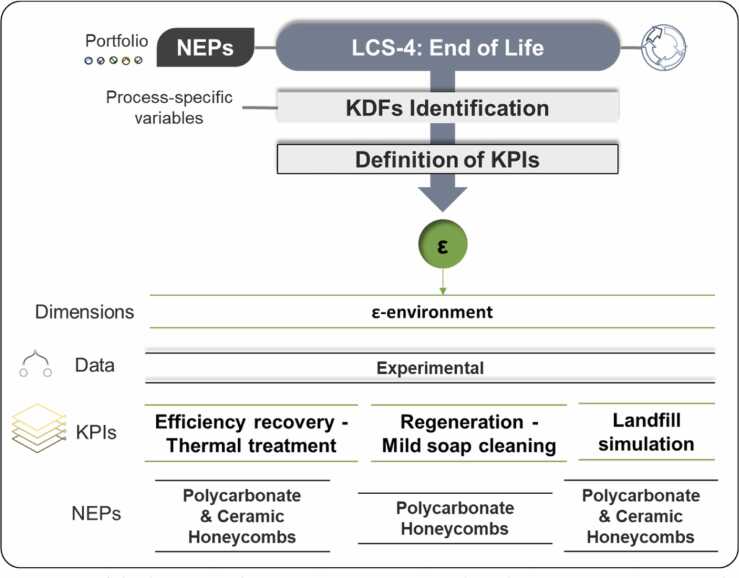
Overview of the NEPs (textile and honeycombs) use phase (LCS-3). This phase encompasses three dimensions: φ-functionality, σ-human health safety and ε-environment. PMMA panels were not considered for further release experiments during use and end-of-life stages.

The assessment during the use phase focuses on the functionality of NEPs and the potential human and environmental exposure resulting from their release during use (such as through abrasion, washing, or operability) (Fig. 3).

#### 2.3.1 φ-functionality dimension

Following functionality, ensuring the quality and durability of PES NEPs becomes imperative ensuring long-term reliability and consumer confidence.

**- Textile - Functionality:** A composite KPI reflecting the photocatalytic efficiency, technical quality, and durability of NEPs is proposed (Table S10). This KPI includes the following elements: i) photocatalytic efficiency with laboratory analysis conducted according to photodegradation tests by assessing the depletion of Nitrogen Oxide (NO) and/or nitrogen dioxide (NO_2_) gases under visible light activation, using chemiluminescence to evaluate the abatement of such gaseous pollutants. NEPs must achieve a photocatalytic efficiency of > 90 % to proceed to quality and stability tests ii) washing and abrasion stability tests: if the NEPs meet the photocatalytic efficiency threshold, they undergo further tests to ensure quality and stability (abrasion resistance following an adaptation of ISO 105 ×12:2016 - Part X12: Colour fastness to rubbing, and EN ISO 105-C06 A1S for washing stability). The percentage of titanium (Ti) remaining on the substrate after these cycles that retained functional performance, was evaluated using ICP-MS and selected as the KPI reflecting the functionality dimension.

#### 2.3.2 σ-human health safety dimension

The potential release of TiO_2_-N NMs is a risk factor that must be addressed during the use-phase. Koivisto, Trabucco [32] investigated the release of TiO_2_ under laboratory-simulated operability from a commercially available photocatalytic air purifier, "Gearbox Wivactive," which consists of ceramic honeycombs coated with TiO_2_-N for air filtration under UV radiation. The impact of the Gearbox Wivactive on indoor concentration levels under RWC conditions was predicted using release factors and a well-mixed indoor aerosol model. This approach to air monitoring during the air filtration process ensures a understanding of potential emissions from the NEPs, contributing to consumers protection.

-**Ceramic Honeycombs - Consumers Exposure**: Air monitoring campaign was performed during the air filtration process to determine possible release from NEPs. This was used to propose a KPI regarding the use phase (air filtration) (Table S11). The release was calculated in a generalized unit to estimate the emission potential for different photocatalytic surfaces under various operational conditions. Before switching on the GearBox, the aerosol concentration inside the box was measured using an external Condensation Particle Counter (CPC, TSI mod. 3775) and a low-cost optical particle counter Alphasense (OPC-N3) inside the box. Two additional tests involved sampling the air flowed through the GearBox using a pump (Bravo Hplus, Tecora, Italy) and then through an absolute quartz fiber filter to collect any TiO_2_-N NPs detached from the air purifier. The total amount of Ti collected on the filters was determined using inductively-coupled argon-plasma radio quadrupole (iCAP RQ) ICP-MS.

#### 2.3.3 ε-environment dimension

**-Textiles Release:** To estimate the KPI for environmental release of textile NEPs, release rate data were measured according to EN ISO 105-C06 (A1S) and analysed using ICP-MS (Table S12). In this dimension, the PCF contains biological uptake kinetics for NMs assessed following the OECD 317 protocol, particularly concerning the application of biosolids to land. *Enchytraeus crypticus* were exposed to TiO_2_ in soil for 14 days to monitor uptake rates (by mass) in biota, followed by a transfer to clean soil for another 14 days to monitor elimination. This process aimed to assess the potential of NMs to release from NEPs and bioaccumulate in soil biota. Internal Ti concentrations in organisms were measured during both the uptake and elimination phases using ICP-MS. TiO_2_ was used in its manufactured form because soil transformation processes are relatively limited compared to more soluble NMs or scenarios with significant light exposure. Thus, the pristine (P25) particles were considered as proxy for environmentally relevant forms. Background TiO_2_ concentrations in soil can be quite elevated (e.g., close to 1000 mg Ti/kg soil), making it challenging to distinguish between background Ti and Ti from NMs. In the test soil (Lufa 2.2), background concentrations ranged between 473–561 mg/kg. Therefore, exposure concentrations needed to be established well above this range. A pilot study was conducted to determine the concentration of Ti required to detect uptake in exposed organisms.

### 2.4 LCS-4: NEPs EoL

#### 2.4.1 ε-environment dimension

**Fig. 4:**
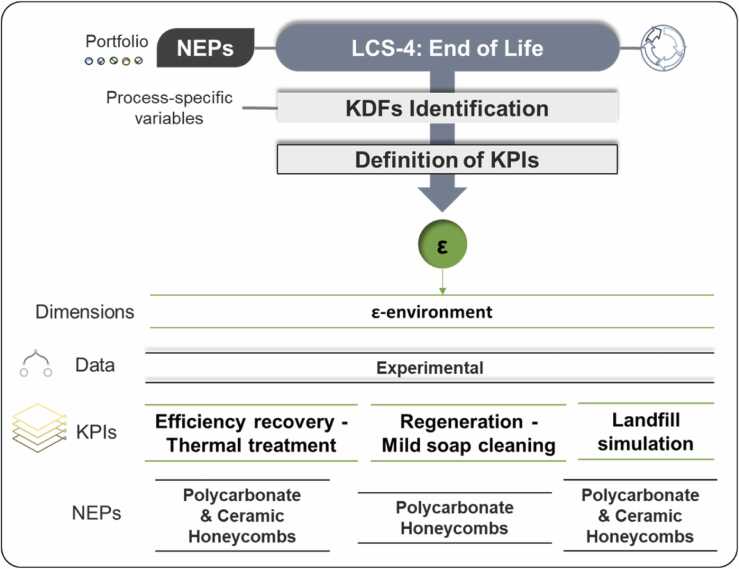
Overview of the honeycombs NEPs EoL (LCS-4). This phase encompasses one dimension: ε-environment. PMMA panels and PES were not considered for end-of-life stages.

Considering the EoL phase of NEPs, it is crucial to define KPIs that address environmental impact, sustainability, and the circular economy. EoL options include materials landfilling presenting different potential emissions to the environment due to leaching when materials are exposed to meteorological agents and the recovery of NMs. To define relevant KPIs, the assessment was focused on honeycombs NEPs provided by industrial partner Colorobbia. Landfill simulations and thermal treatments were performed for ceramic honeycombs, and landfill simulations and mild soap cleaning for polymeric honeycombs, used in air purifier filter devices (Fig. 4).

**- Honeycombs Efficiency Recovery - Thermal Treatment:** The environmental release during efficiency recovery was identified as a critical hotspot for both polycarbonate and ceramic honeycombs. For efficiency recovery, a thermal treatment protocol established by the industrial partner Colorobbia was used (Table S13) ensuring they do not pose environmental hazards post-treatment. The setup included an oven, a scanning mobility particle sizer (SMPS, Nanoscan TSI), an optical particle sizer (OPS TSI), and a cassette housing a TEM grid connected to a pump (SENSIDYNE GilAir Plus) for particle collection.

**-Polycarbonate Honeycombs NEPs regeneration - Mild soap cleaning:** A regeneration washing protocol established by the partner Colorobbia was followed. This method involved washing the NEPs with distilled water and mild soap. The first step was heating distilled water with mild soap under stirring. Once a specific temperature was reached, polymeric honeycombs were placed separately in single plastic containers and filled with the mixture. The honeycombs were shaken every hour until the water returned to room temperature, and a rinsing process was performed. Ti release concentrations in both washing and rinsing waters were determined by ICP-MS (Table S13).

**-Honeycombs - Landfill simulation:** Due to technical limitations for simulating incineration, the release hotspot was investigated by landfilling tests through leaching experiments to simulate the potential release of NMs during the EoL phase from ceramic and polycarbonate honeycombs (Table S13). The experiments followed the EPA Standard Method 1311. Two replicates of used and non-used honeycombs and two blanks were shredded into small pieces. The extraction fluid was prepared by adding glacial acetic acid to deionized water. The solid phase was extracted and samples were rotated end-over-end for several hours. Then, a qualitative solid-liquid separation was performed using a cellulose filter, and aliquots of the leachate solution were characterized by TEM analysis. The total amount of Ti was quantified by ICP-MS (mg/Kg) and converted considering the surface area.

These KPIs provide a comprehensive evaluation of the efficiency recovery, regeneration, and landfill simulation for ceramic and polycarbonate honeycombs NEPs, that resulted aligned with environmental safety and sustainability goals.

### 2.5 MultiOptimal360™ applied to TiO_2_NMs from synthesis to incorporation and use phase

The goal of the optimal multi-performance design cases (multi-optimal solutions) for the synthesis of safe and sustainable TiO_2_ NMs is to identify the combination of KDFs levels that simultaneously fulfil the design criteria of the identified KPIs, which include: minimizing environmental impacts (represented by associated CO_2_ emissions), maximizing NMs photocatalytic efficiency (RhB model consumption), and reducing TiO_2_ NMs synthesis costs. The case study includes the comparison among the design alternatives, relevant to the project objectives. Design alternatives associated to the LCS-1 define the first KDF-1 allowing for five discrete values or levels associated to the specific photocatalytic NM investigated:

- TAC: Commercial TiO_2_ NMs in suspension 3 wt% (supplier Colorobbia)
- TiO_2_ @SiO_2_: Composite material obtained by heterocoagulation based TAC and SiO_2_, synthesised through 24-hour ball milling process.
- TiO_2_ DT51‐US: Commercial TiO_2_ powder (supplier CristalACTiV™) obtained after 1 h of sonochemical ultrasound treatment.
- TGO: Composite material based on TiO_2_ DT51 powder supported on graphene oxide, after 1 h of sonochemical ultrasound treatment.
- g‐C3N4: synthesized carbon nitride powder material obtained from melamine through thermally heating treatment.

The scope of the quantitative case study encompasses the analysis of the synthesis, incorporation and use phase. The NMs differ on the synthesis methods and the testing phase, which simulated the use phase, involved varying exposure times. The incorporation phase remained consistent across all materials. Consequently, the KDFs are referred only to the LCS-1 and LCS-3. Otherwise, all KPIs account for the whole lifecycle. LCS4- was beyond the scope of this case study. To achieve the objective, a multi-optimization algorithm based on MCDA was implemented. The algorithm operates on a harmonized dataset derived from various inputs associated with the design alternatives. .

**Table 1:** KPIs-KDFs-PCFs to obtain the set of candidates multi-optimal SSbD solutions.

**Case study**	TiO_2_**NMs synthesis process**
**Goal**	Identify the conditions for NMs synthesis (find values of KDF1 and KDF2) that simultaneously comply with the following design **criteria**: 1. Synthesize TiO_2_ NMs displaying highest photocatalytic functionality 2. Minimize synthesis process costs 3. Minimise CO_2_ emissions per unit synthesized NM 4. Minimize human toxicity potential (mid-point indicator) associated to synthesis process
**Scope**	Analysis of the synthesis process in a “cradle to gate” approach. **System**: TiO_2_ NMs synthesis **System boundaries**: from raw materials and energy sources to the delivery of product unit **Functional Unit**: unit of 5 g synthesized TiO_2_-NMs
KPIs	**KPI 1:** photocatalytic efficiency (moles of models consumed) **KPI 2:** functional unit cost. **KPI 3:** global warming potential per functional unit **KPI 4**: human toxicity potential per functional unit
composite KPIs	- **cKPI1:** functional performance (pollutant abating efficiency) per unit cost (including NMs filter CAPEX cost of synthesis and incorporation of NMs and OPEX cost represented by energy use for operating the filtering element)
KDFs	**KDF 1:** type of nano-photocatalyst (associated to LC1-synthesis) **KDF 2:** irradiation time (in minutes), critical for activating the photocatalytic properties and for the energy consumption (associated to LC3-use phase) **KDF 3**: irradiation source (visible sunlight and UV-light lamp), (associated to LC3-use phase)
PCF	**PCF1:** TOF, calculated as the initial concentration of the reagent per efficiency achieved at time t (in seconds), divided by the moles of active phase. This quantifies catalytic activity per amount of catalyst over time. **PCF2:** Chemical Oxygen Demand (COD) as an indicator of water pollution and represents the quantity in mg of oxygen necessary to chemically oxidize both organic and inorganic pollutants present in a litre of water. This is possible with photoluminescence techniques or by incubating photocatalysts with an •OH scavenger such as nitroaniline.
Data generation and assessment criteria	**Measurement method:** Decision space data generation - KDF1- identification of nano-photocatalyst **(**discrete variable). - KDF2 - chronometric measurement of irradiation time with chrono gauge 0 −3600 s, sensitivity= 100 s^−1^ (continuous variable). - KDF3. irradiation source: definition if exposure to natural sun light or to artificial light (UV lamp) (discrete variable). Performance space data generation: - KPI1 - Abatement of pollutants in use phase, Laboratory testing under specific irradiation sources, involving the degradation of RhB dye, quantified by UV-Vis absorption analysis. (continuous variable) - KPI2 - LCC of synthesis and use phase including reagents and energy use, according to the gross energy requirement (GER). As regards energy costs, a value of 0.13 € / KWh was considered (updated to the year 2021). In our study the variable "cost" was chosen as the control function (f‐c) (continuous variable) - KPI3- environmental impact LCA for global warming potential assessment impact category (mid-point assessment). The environmental impact data were obtained with the Ecoinvent v.3.7 database and with the Open LCA software. The method of use remained the CLM 2001 and the CO_2_ emissions in Kg ‐ equivalents per KWh of electricity were equal to 0.41615 (Kg CO_2_ ‐ eq / kWh). (continuous variable) - KPI4 LCA for human toxicity potential impact category (mid-point assessment), LCA the human toxicity impact data were obtained with the Ecoinvent v.3.7 database and with the Open LCA software. The impact method used is CLM 2001 (continuous variable) **Thresholds/target values and criteria:** − Areas with photocatalytic efficiency > 60 % are considered acceptable. − Maximisation of cKP I1: functional performance per unit cost − Minimisation of KPI 3- environmental impact, and KPI4: toxicity level for humans

**Fig. 5:**
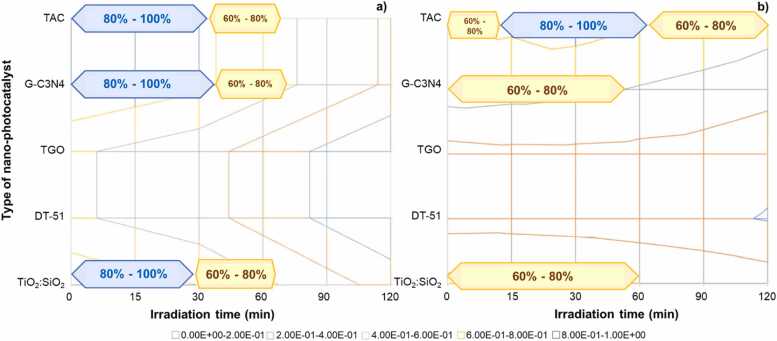
Decision space for the KDF1-nano-photochatalist type, KDF2- irradiation time, (KPI 3 option selected is set to irradiation by UV lamp).

The Fig. 5 two diagrams show the: Set of sustainable by design options represented in decision space referred to KPI2 economic only, which yields best solutions for low irradiation times (Fig. 5***a**)*, and the set of sustainable by design options represented in decision space referred to environmental, safe and composite-functional cKPI showing that TiO_2_-SiO_2_ photocatalyst provides multiperformance level I the range of 60 %−80 % of the maximum attainable (Fig. 5***b**)*. The multiperformance factor considered implies weighting 30 % the compound functional factor (cKPI) and 70 % the environmental (and safety) KPIs.

The MultiOptimal360TM IT platform^5^ was employed for the.

• Phase III - impact assessment, to (a) investigate the implications of using various nano-photocatalysts options in association to their synthesis processes, photocatalyst options, source and exposure time, (b) assess the dependence of functional efficiency in relation to different photocatalyst options, irradiation sources and exposure times, and (c) to assess the mid-point human toxicological and environmental impacts associated with photocatalyst options, irradiation sources and exposure times.
• Phase-IV - interpretation and identification of SSbD solutions - to identify the set of candidates SSbD cases based on KPIs performance levels within the range of 60 %−80 % of the highest performance obtainable within the range of KDFs options (decision space) (see Fig. 5).

The human centric decision process starts by considering the set of multi-optimal design options. Other combinations of KPIs with specific objectives can be found in [33] where in the Fig. 1 of the [33] paper, the authors show the SSbD approach related to materials and processes and which KPIs objective can be used in a holistic manner according to the data generated.

MultiOptimal360™ allowed also correlating the set of multi-optimal synthesis KDFs values to the corresponding NPs expected PCFs and to the corresponding KPIs values, providing a comprehensive scenario quantitatively described with a selected set of SSbD optional solutions.

## 3 Discussion

### 3.1 Towards a holistic methodological approach to SSbD in nanotechnology

This roadmap details the methodology deployed in the ASINA project, aimed at advancing and implementing SSbD criteria within nanomanufacturing. A common understanding of SSbD principles is fundamental for the development and successful implementation of SSbD framework and communication activities in this field [34]. The roadmap represents a synthesis of multiple ASINA components: the ASINA-DST, ASINA-SMM, the ASINA-AOP testing strategy, DT technologies, pilot actions, ML applications and new approaches and methodologies considered/proposed throughout the LCSs. By integrating these elements, this report encapsulates a holistic assessment approach providing methods to measure all relevant factors within specific case studies. Adopting the structured approach outlined in this roadmap lays a robust foundation for the effective dissemination of future results to ensure consistency and transparency in reporting outcomes, facilitating the continuity and reproducibility of subsequent studies. It provides a common language and set of metrics that can be applied, simplifying the communication of complex technical information to a broader audience, including policymakers and the scientific community. A discussion on the i) impact of structured quantitative elements on SSbD integration with AI, the challenges associated with the KPIs definitions, can be found here [13].

### 3.2 Towards a common harmonized roadmap

The proposed roadmap aims to foster a collaborative environment that supports the efforts towards a common SSbD roadmap, ultimately aligning with the European Union's ambitious Green Deal objectives. The case study on nano-silver-based antimicrobial textiles [13] follows the same innovative methodology as outlined in this paper. To accelerate the transition towards a unified SSbD roadmap based on different target industrial sectors, we provided a table (Table 2) that captures the main differences and commonalities between the case studies. This comparison highlights the methodological approach needed to define KPIs (tools, measurements, laboratory testing) and facilitates knowledge transfer across industry sectors. The proposed SSbD methodology leaves the freedom to prioritise the assessment dimensions (e.g. safety over functionality or cost) but it does not require it as its the methodological power resides in the simultaneous assessment of all KPIs to find multi-optimal SSbD solutions. The dimensions prioritisation and KPIs importance ranking becomes an implicit procedure when sorting or ordering the multi-optimal design options, emphasizing the human centric approach in decision making. Table 2 captures the dual aspects of (i) the methodology and approach for quantitative assessment of each dimension and (ii) the quantitative elements along the dimensions.

**Table 2:** Intercomparison of case studies: methodological approaches and quantitative elements across lifecycle and SSbD dimensions (φ-functionality, σ-human health safety, ε-environment and γ-cost).

**Case-study 1 (CS-1): Nano-Silver-based Antimicrobial Textile Coatings** VS **Case-study 2 (CS-2): Nano- Titanium Dioxide -based Photocatalytic depolluting air filters, textiles, and honeycombs Coatings**		
LCS−1: Synthesis phase		
Commonalities		**Differences**
φ	-Both studies begin with a hypothesis rationale, which facilitates the development of an SSbD NMs portfolio to undergo comprehensive SSbD assessment. -Laboratory experiments are required to define the functionalities of the NMs portfolio in both case studies.	-CS−1 utilizes a tailored Design of Experiment (DoE) approach to systematically investigate different tiers of SSbD options, taking into account synthesis parameters (KDFs). CS−2 is grounded on a hypothesis-driven rationale without employing a DoE. The KDFs focus on selecting appropriate surface modifiers and/or composite composition NMs. -Different KPIs, KDFs, and PCFs are used in each case study. CS−1: Antimicrobial performance. CS−2: Photocatalytic efficiency.
σ	Not assessed	
ε	-Both studies employ the same methodology and approach for KPIs, adhering to EN ISO 14044:2006. -Data from the Ecoinvent Database v3.7 for the Open LCA software.	-The approach varies depending on the synthesis process-related data, energy requirements, and materials (raw materials and chemicals involved in the process) for the definition of the functional unit.
γ	-Both case studies utilize the same methodology and approach for KPIs concerning LCC, based on the ISO 15686 −5. -This approach includes representative costs associated with capital goods and energy use per NM unit quantity (batch), measured in kilograms	-Different KDFs are considered, reflecting the specific materials and associated costs involved in their respective synthesis processes. For example, CS−1: concentration of HEC (hydroxyethyl cellulose) and NaOH (sodium hydroxide) and their associated costs VS CS−2: titanium tetraisopropoxide, ammonium hydroxide etc.,
LCS−2: NMs incorporation phase		
φ	-Both case studies utilize the same methodology and approach, involving laboratory experiments to define the minimum amount of NM required to achieve the desired functionality, using ICP-MS. -The same spray coating technique is employed to develop the NEPs.	-NEPs developed differ between the two case studies, reflecting the specific applications and materials used (Textile NEPs in CS−1 and honeycombs and PMMA panels in CS−2).
σ	-Both case studies employ the same methodology for assessing occupational exposure, a tiered assessment incorporating monitoring campaigns, laboratory analysis of air particles, and probabilistic modelling. This approach[26], [29], estimates workers' exposure levels and safety, targeting the 95th percentile of the lognormal distribution of 8-hour exposure to NMs. -Similarly, the inhalation risk assessment KPI utilizes a multi-tier NAM, integrating emission campaigns, MPPD modelling, dose translation, and in vitro testing[28]. -The same spray coating technique is employed to develop the NEPs. -In both case studies, DT technology is explored. -Occupational exposure levels assessed are aligned with established safety guidelines within recommended standards requirements.	-Different target values are based on recommended standards, with comparisons made against the OEL. In our case, the NIOSH REL as an 8-hour TWA for respirable fraction. -In CS−2 the BoD concept expressed in DALYs was established to estimate health impacts from industrial NM emissions, of the potential public health impacts from TiO_2_-N exposure. However, in CS−1, the exposure response functions were not available.
ε	-Both case studies utilize a comprehensive approach for environmental impact assessment, employing emission monitoring campaigns, a mechanistic model analysing mass flows associated with Local Exhaust Ventilation (LEV) filters[29], and a single compartment model (bi-Gaussian plume model) to estimate the accumulation concentrations of NMs in the soil top layer[30].	-Different target values used in both case studies in a 10-year production scenario.
γ	-Both case studies utilize the same methodology and approach for KPIs concerning LCC, based on the ISO 15686 −5 methodology.	In CS−1 spray coating process at WITEK was used as the basis for representative cost assessment. In CS−2, the pilot action CeNTI was considered, which includes differences in KDFs and energy requirements of the systems.
LCS−3: NEPs use phase		
φ	-Laboratory experiments are required to define the functionalities and quality (washing stability and abrasion resistance in case of textiles) of the NEPs portfolio in both case studies.	-Different KPIs are used in each case study. CS−1 Antimicrobial performance CS−2 NMs Photocatalytic efficiency.
σ	-	-CS−1 employs a tiered approach to assess dermal exposure, incorporating abrasion measurements, permeability modelling to estimate intake mass, and skin irritation test using artificial sweat and reconstructed human epidermis models. -CS−2 focuses on consumer exposure estimates through air emission monitoring to determine possible release and predictive modelling of TiO_2_ exposure in indoor environments.
ε	Biological fate uptake kinetics parameters focusing on bioaccumulation in soil invertebrates *E. crypticus* were exposed to NMs for 14 days to monitor uptake rates (by mass) in biota, followed by a transfer to clean soil for another 14 days to monitor elimination.	In CS−1, a composite KPI showcasing the Predicted Environmental Concentrations using SEAT (Simple Environmental Exposure Assessment Tool), SimpleBox4nano, and NanoFASE (Nanomaterial Fate and Speciation in the Environment), by using washing water release concentration. In CS−2 an environmental KPI was not defined. Solely the release potential.
γ	Not assessed	
LCS−4: NEPs EoL		
φ	Not assessed	
σ		
ε	Both case studies assess the leaching potential NEPs based on a soft abrasion test using the standardized protocol EPA Standard Method 1311 to simulate end-of-life conditions under landfilling scenarios.	Additional evaluation in CS−2 which include steps for efficiency recovery (Ceramic honeycombs) through thermal treatments and NEP regeneration (Polycarbonate honeycombs) using mild soap cleaning.
γ	Not assessed	
Lifecycle inherent safety: NMs intrinsic hazard properties		
σ	-In both case studies, an AOP strategy was followed for defining a hazard testing strategy. -KDFs contain exposure condition and experimental parameters. -PCFs include pchem properties measured in the cellular media -similar assays were used overall with few differences among the subsequent KPIs.	In CS−2 the interaction between cells and NMs was analysed using side scatter (SSC) values obtained by cytofluorimetric analyses. These SSC values served as a proxy for cell and NM interaction and uptake, and were used in the inhalation KPI. - In CS−2, the NMs did not undergo a gastrointestinal simulation method before being tested in vitro. - In CS−2 a dermal KPI is included. In CS−1, the dermal KPI was integrated with modelling in the use phase of NEPs. - In CS−2 the NMs hazard focused on evaluating oxidative stress by detecting a set of indicators under acellular conditions.
ε		-In CS−1, zebrafish (*Danio rerio*) was used to define an aquatic hazard KPI and potworms *Enchytraeus crypticus* for the soil bioaccumulation. No alternative in vivo experiments were performed in CS−2.

## 4 Conclusions

This roadmap shows in detail how to apply SSbD criteria to nanomanufacturing. The most important aspects of this roadmap are outlined below:

• It offers quantitative metrics and thorough detailed guidelines promoting the SSbD of two highly significant applications for environmental protection: antimicrobial textiles and photocatalytic air filters.
• It incorporates multifaceted dimensions of SSbD to foster a holistic lifecycle quantitative assessment, which also establishes a generic basis for future SSbD implementations and comparisons with SSbD studies already carried out.
• It serves as consensus prototypes towards a common SSbD roadmap, ultimately aligning with the EU Union's ambitious Green Deal objectives. A cross comparison among industrial sectors and case studies reveals key similarities and differences of the roadmap, in order to align efforts.
• It integrates a DST system, specifically the MultiOptimal360™ IT platform, which identifies the range of design options that can optimize the required performance within a multidimensional space.
• Additionally, it incorporates cutting-edge technologies like Digital Twins (DT) to enhance the design and manufacturing of NEPs, leading to safe and sustainable production processes.

The established methodology is available for use by other researchers and innovators in the field. As we advance the work initiated by the ASINA project, ongoing collaboration between academia, industry, and regulatory bodies will be essential for further refining these approaches. This roadmap, indeed, invites the researchers and industrial communities to engage with, critique, and further develop and improve its practical and impactful approach in product design and development.

## CRediT authorship contribution statement

**Joonas Koivisto:** Validation, Investigation, Formal analysis, Data curation. **Elma Lahive:** Methodology, Investigation, Data curation, Conceptualization. **Valentina Dami:** Investigation, Data curation. **Juliana Oliveira:** Writing – review & editing, Investigation, Data curation, Conceptualization. **Andrea Cioni:** Investigation, Data curation. **David Burrueco-Subirà:** Methodology, Investigation, Data curation, Conceptualization. **Massimo Perucca:** Writing – review & editing, Methodology, Formal analysis, Conceptualization. **Socorro Vázquez-Campos:** Validation, Methodology, Conceptualization. **Giovanni Baldi:** Data curation. **Alessia Nicosia:** Validation, Methodology, Investigation, Data curation. **Fabrizio Ravegnani:** Investigation, Data curation. **Irini Furxhi:** Writing – review & editing, Writing – original draft, Methodology, Data curation, Conceptualization. **Anna Luisa Costa:** Writing – review & editing, Validation, Supervision, Methodology, Investigation, Funding acquisition, Formal analysis, Data curation, Conceptualization. **Giulia Motta:** Methodology, Investigation, Data curation. **Marie Carriere:** Methodology, Investigation, Data curation. **Patrick Cronin:** Methodology, Investigation. **Rossella Bengalli:** Writing – review & editing, Investigation, Data curation, Conceptualization. **Magda Blosi:** Validation, Methodology, Investigation, Data curation, Conceptualization. **Paride Mantecca:** Validation, Data curation, Conceptualization. **Jesus Lopez de Ipiña:** Validation, Methodology, Investigation, Data curation, Conceptualization.

## Author Statement

Project s.a.s. of Massimo Perucca, owner of the MultiOptimal360TM IT platform (https://www.projecthub360.com/) developed the DST as an integral part of the project. No potential conflict of interest was reported by the authors

## Declaration of Competing Interest

The authors declare that they have no known competing financial interests or personal relationships that could have appeared to influence the work reported in this paper.

## Footnotes

Appendix A Supplementary data associated with this article can be found in the online version at doi:10.1016/j.csbj.2024.10.001.

## Footnotes

1 https://www.ami2030.eu/wp-content/uploads/2023/04/Ami2030-Dossier-2.pdf

2 *nano-enabled definition according to ISO/TS 80004–1:2015: exhibiting function or performance only possible with nanotechnology (*https://www.iso.org/obp/ui/#iso:std:iso:ts:80004:−1:ed-2:v1:en:sec:2.15*)*

3 https://www.nanosafetycluster.eu/nsc-overview/spg/#1706188945110-faa40a78-da72

4 ISO 15686–5:2017 Buildings and constructed assets — Service life planning — Part 5: Life-cycle costing

5 https://www.projecthub360.com/
