# Supplementary material for: Advancing titanium dioxide coated photocatalytic depolluting surfaces: Leveraging ASINA's roadmap for safer and sustainable solutions
Source: Comput Struct Biotechnol J. 2024 Oct 17;25:269–80. doi: 10.1016/j.csbj.2024.10.001 (PMC11576338; doi:10.1016/j.csbj.2024.10.001)
Supplement: Supplementary file 1 — Supplementary material [file mmc1.docx]

**SUPPLEMENTARY MATERIAL**

**A Roadmap Towards Safe and Sustainable by Design Nanotechnology: Titanium Dioxide Coated Photocatalytic Depolluting Surfaces Production by the ASINA know-how.**

Irini Furxhi^1^, Massimo Perucca*^2^, Giovanni Baldi^3^, Valentina Dami^3^, Andrea Cioni^3^, Antti Joonas Koivisto^4,5,6^, Rossella Bengalli^7^, Paride Mantecca^7^, Giulia Motta^7^, Marie Carriere^8^, Alessia Nicosia^9^, Fabrizio Ravegnani^9^, David Burrueco-Subirà^10^, Socorro Vázquez-Campos^10^, Elma Lahive^11^, Jesús Lopez de Ipiña^12^, Juliana Oliveira^13^, Patrick Conin^14^, Magda Blosi^1^, Anna Costa*^1^.

*^1^ CNR-ISSMC Istituto di Scienza e Tecnologia dei Materiali Ceramici, Via Granarolo, 64, 48018 Faenza, RA, Italy.*

*^2^ Project HUB360 C.so Laghi 22, 10051 Avigliana - Metropolitan city of Turin, ITALY*

^3^ *COLOROBBIA Consulting srl, Via Pietramarina 53, 50059 Sovigliana-Vinci, Fi, Italy*

*^4^ APM Air Pollution Management, Mattilanmäki 38, FI-33610 Tampere, Finland*

*^5^ INAR Institute for Atmospheric and Earth System Research, University of Helsinki, PL 64, FI-00014 UHEL, Helsinki, Finland*

*^6^ ARCHE Consulting, Liefkensstraat 35D, Wondelgem B-9032, Belgium.*

*^7^ POLARIS Research Center, Dept. of Earth and Environmental Sciences, University of Milano Bicocca, Piazza della Scienza 1, 20126, Milano, Italy.*

*^8^ CEA Univ. Grenoble Alpes, CNRS, Grenoble INP, IRIG, SYMMES, Grenoble 38000, France*

*^9^ CNR-ISAC Institute of Atmospheric Sciences and Climate, Via Gobetti 101, 40129 Bologna, Italy*

*^10^ LEITAT Technological Center, C/ de la Innovació 2, 08225 Terrassa, Barcelona, Spain.*

*^11^Centre for Ecology & Hydrology (UKCEH), United Kingdom, England*

*^12^ TECNALIA Research and Innovation - Basque Research and Technology Alliance (BRTA), Parque Tecnológico de Alava, Leonardo Da Vinci 11, 01510 Miñano, Spain.*

*^13^ CeNTI - Centre of Nanotechnology and Smart Materials, Rua Fernando Mesquita 2785, 4760-034 Vila Nova de Famalicão, Portugal*

*^14^MicaNanotech Ltd, Limerick, Ireland*

*Correspondence: [anna.costa@issmc.cnr.it](mailto:anna.costa@issmc.cnr.it), massimo.perucca@project-sas.com

Irini Furxhi: [irini.furxhi@issmc.cnr.it](mailto:irini.furxhi@issmc.cnr.it)

Antti Joonas Koivisto: joonas.apm@gmail.com

Rossella Bengalli: ([rossella.bengalli@unimib.it](mailto:rossella.bengalli@unimib.it)

Paride Mantecca: [paride.mantecca@unimib.it](mailto:paride.mantecca@unimib.it)

Giulia Motta: [giulia.motta@unimib.it](mailto:giulia.motta@unimib.it)

Alessia Nicosia: [a.nicosia@isac.cnr.it](mailto:a.nicosia@isac.cnr.it)

*Fabrizio Ravegnani: f.ravegnani@isac.cnr.it*

David Burrueco-Subirà: dburrueco@leitat.org

Socorro Vázquez-Campos: [svazquez@leitat.org](mailto:svazquez@leitat.org)

Elma Lahive: [elmhiv@ceh.ac.uk](mailto:elmhiv@ceh.ac.uk)

Magda Blosi: [magda.blosi@issmc.cnr.it](mailto:magda.blosi@issmc.cnr.it)

Jesús Lopez de Ipiña: jesus.lopezdeipina@tecnalia.com

Juliana Oliveira: jaoliveira@centi.pt

Marie Carriere: [marie.carriere@cea.fr](mailto:marie.carriere@cea.fr)

Giovanni Baldi: [baldig@colorobbia.it](mailto:baldig@colorobbia.it)

Valentina Dami: [damiv@colorobbia.it](mailto:damiv@colorobbia.it)

Andrea Cioni: *[cionia@colorobbia.it](mailto:cionia@colorobbia.it)*

Patrick Conin: [Patrickcronin4@gmail.com](mailto:Patrickcronin4@gmail.com).

# Results and Outcomes

This report demonstrates in detail a case study within the ASINA project, aimed at instantiating a roadmap with quantitative metrics for Safe(r) and (more) Sustainable by Design (SSbD) options, starting with a description of ASINA’s roadmap across the product lifecycle, outlining the quantitative elements of our methodology: Physical-Chemical Features (PCFs), Key Decision Factors (KDFs), and Key Performance Indicators (KPIs).

In the following chapters, a high-level overview of the technical aspects involved in defining KPIs, KDFs, and PCFs including design rationales, experimental procedures, and tools/models used across the entire NMs lifecycle is provided, addressing all relevant dimensions.

## LCS-1: Synthesis phase

### φ-functionality dimension

Table S1. A structured overview of NMs Functionality KPI of φ-Functionality dimension in LCS-1.

| **NMs Functionality KPI:** A simple KPI assessing the photocatalytic efficiency of pristine NMs in suspension.  **-Measurement method:** Laboratory testing under specific irradiation sources, involving the degradation of organic pollutants such as Rhodamine B (RhB) dye, which serves as a reaction model. The reduction in RhB is quantified by analysing its absorbance spectra through UV-Vis absorption analysis. **Unit of measurement:** Percentage (%). **Ranges:** 0-100%. **Thresholds/target values:** Determined through expert judgment.  **-Objective:** KPI maximization. | |
| --- | --- |
| KDFs | **KDF 1:** modification agent (uncoated/non-doped, N-doped, SiO_2_-coated, N-doped and SiO_2_-coated).  **KDF 2:** exposure dose (mg/L) and exposure duration (minutes), which influence the interaction extent between the NMs and pollutants.  **KDF 3:** irradiation source (types include visible light and UV-light) and duration (minutes), critical for activating the photocatalytic properties |
| PCFs | PCF 1: primary particle size (nm) via Transmission Electron Microscopy (TEM)(OECD 2023).  PCF 2: hydrodynamic diameter (z-average) and its polydispersity index (PdI) (nm) and particle size distribution of suspensions by Dynamic Light Scattering (DLS), according to ISO 22412:2017.  PCF 3: ζ-potential (mV) utilizing Electrophoretic Light Scattering technique (ELS) in suspension, in accordance with ISO 22412:2008E.  PCF 4: specific surface area (m^2^/g) determined by Brunauer – Emmett – Teller (BET) analysis  PCF 5: band gap (eV), measured using UV/VIS/NIR spectrophotometry. The reflectance percentage (R) relative to the wavelength (nm) is processed through the Kubelka-Munk equation to obtain the Tauc plot, a graphical method aiding in understanding the optical properties.  PCF 6: crystalline phases determined through crystallographic characterization by X‐Ray Diffraction (XRD) analysis  PCF 7: turn over frequency (TOF), calculated as the initial concentration of the reagent per efficiency achieved at time t (in seconds), divided by the moles of active phase. This quantifies catalytic activity per amount of catalyst over time.  PCFs are indicative and could contain a variety of features depending on the specific requirements and applications of the NMs. In addition to the above, (i) quantification of the atomic concentrations of elemental compositions derived by X-ray photoelectron spectroscopy (XPS) analysis and (ii) crystallographic structure-related information by X-ray diffraction analysis (XRD) and iii) TEM images to determine agglomerates of NMs were collected. |

### ε-environment dimension

Table S2. A structured overview of NMs synthesis sustainability KPI of ε-environment dimension in LCS-1.

| **NMs synthesis sustainability:** A composite KPI that assesses synthesis process contributions to global warming through greenhouse gas emissions.  **-Measurement method:** Cradle to gate modelling approach following EN ISO 14044:2006. Data from Ecoinvent Database v3.7 for Open LCA software by applying CML 2001 impact assessment method. The defined functional unit is the amount electricity for the synthesis of the technical unit associated to 5 g of TiO_2_ NMs.  **Unit of measurement:** kilograms of carbon dioxide equivalent (kg CO_2_ eq). **Ranges:** Determined per each case-study based on modelling parameters. **Thresholds/target values:** A process is considered carbon neutral if the CO_2_ amount captures equally the other fossil emissions.  **-Objective:** KPIs minimization. | |
| --- | --- |
| KPIs | Categorized into three subcategories based on emission sources: (1) fossil resources, (2) land use change, and (3) bio-based resources.  **KPI 1:** fossil CO_2_ emissions per unit product (kg CO_2_ eq)  **KPI 2:** CO_2_ eq from land transformation (kg CO_2_ eq)  **KPI 3:** Biogenic CO_2_ eq (kg CO_2_ eq)  **KPI 4:** CO_2_ uptake (kg CO_2_ eq) |
| KDFs | **KDF 1:** modification agent (uncoated/non-doped, N-doped, SiO₂-coated, N-doped and SiO₂-coated).  **KDF 2:** exposure dose (mg/L) and exposure duration (minutes).  **KDF 3:** irradiation source (visible light and UV-light) |

### σ-human health safety dimension

For identifying Adverse Outcomes (AOs) and underlying mechanisms, relevant KPIs were established to monitor the potential toxicological impacts of TiO_2_ NMs. Firstly, to organize the mechanistic information on TiO_2_ NM toxicity, and following recommendations of OECD WPNM NanoAOP project, the literature was reviewed to identify the most frequently reported AOs and toxicity mechanisms after dermal, pulmonary, or intestinal exposure to TiO_2_ NMs in experimental animals or *in vitro* cells. Secondly, AOPwiki database was searched for AOPs in which TiO_2_ is defined as a stressor, as well as AOPs reflecting its toxicity mechanisms. This search included Molecular Initiating Events (MIEs), Key Events (KEs), and AOs related to these toxicity mechanisms. The literature and AOPwiki were both consulted because they contain complementary information: some putative AOPs are reported in the literature but are not yet included in AOPwiki, and some AOPs listed as "in development" in AOPwiki have not yet been published in peer-reviewed journals. This comprehensive approach ensures that the full range of potential toxicological mechanisms and pathways associated with different types of TiO_2_ NMs is captured, including those with surface modifications or doping.

Table S3. High level results summary of ASINA-AOP testing strategy for evaluating NM safety by linking molecular-level interactions to adverse health outcome.

| **AOPs findings** | |
| --- | --- |
| **🡪**The first AOP, merges two existing AOPs: | |
| AOP144 [*Endocytic lysosomal uptake leading to liver fibrosis*] | Describes how MIE leads to liver fibrosis. NMs are listed as stressors. Therefore, applying this AOP scheme to describe TiO_2_ NMs toxicity mechanisms is relevant. |
| AOP34 [*LXR activation leading to hepatic steatosis*] | Describes a series of KEs that link TiO_2_ endocytic lysosomal uptake (MIE) to liver fibrosis (AO1), liver oedema (AO2) and steatosis (AO3) (Brand *et al.* 2020). AOP34 does not mention NMs as stressors, but a KE and two AOs have been included in the AOP, based on expert judgement and recently published data (Gerloff *et al.* 2017). |
| **🡪**The second AOP has been proposed by (Braakhuis *et al.* 2021) to describe how cellular uptake of TiO_2_ NMs in the intestine (MIE) leads to intestinal tumours (AO). The series of KEs linking the MIE and the AOP encompass oxidative stress, inflammation, proliferation of intestinal cells and DNA damage, which are toxicity mechanisms frequently reported to explain TiO_2_ NMs toxicity. | |
| **🡪**Other AOPs from AOPwiki have been considered: | |
| AOP208 [*Janus kinase (JAK)/Signal transducer and activator of transcription (STAT) and Transforming growth factor (TGF)-beta pathways activation leading to reproductive failure*] | |
| AOP282 [*Adverse outcome pathway on photochemical toxicity initiated by light exposure*] | It includes two KEs that lead to the AO-reproductive failure. No MIE is indicated. The putative AOP describes how photoactivation of the stressor leads to inflammation via oxidation of lipids/proteins, which is relevant to the mechanisms of TiO_2_ NMs toxicity in the literature. |
| The two AOPs above describe cellular pathways that are activated by photoactive stressors. AOP208 includes UV-activated TiO_2_ NMs as a stressor and was developed to describe their toxicity towards nematode *Caenorhabditis elegans.* AOP282 is initiated by photoreactive molecules when they are exposed to light. | |
| **Key Events** | |
| **🡪KE TGF-beta pathway** | |
| **AOP206** [*Peroxisome proliferator-activated receptors γ inactivation leading to lung fibrosi*s] | |
| **AOP347** [*Toll-like receptor 4 activation and peroxisome proliferator-activated receptor gamma inactivation leading to pulmonary fibrosis*], which both lead to the AO lung fibrosis. | |
| **🡪KE Oxidative stress** | |
| AOP207 [*NADPH oxidase and P38 MAPK activation leading to reproductive failure in Caenorhabditis elegans*]. | The MIE is NADPH oxidase activation that leads to the KEs ROS formation, DNA damage-repair, HIF-1 activation, mitochondrial damage, apoptosis. |
| AOP210 [*Activation of c-Jun N-terminal kinase (JNK) and FOXO and reduction of WNT pathways leading to reproductive failure: Integrated multi-OMICS approach for AOP building*], | Was identified because it shares the common KE oxidative stress, which is also KE of AOP260 and the common AO reproductive failure, which is also the AO of AOP208.  In AOP210, no MIE is proposed. AOP210 includes a series of KEs that are oxidative stress, activation of JNK, activation of FOXO, reduction of WNT signalling and defect in embryogenesis. |
| The stressors proposed for AOP207 and AOP210 are NMs. Therefore, it is proposed that TiO_2_ NMs could be also defined as a stressor. Indeed, the MIE of AOP207 is NADPH oxidase activation, and TiO_2_ NMs have been described as inducing this enzyme and as triggering most of the KEs of this AOP (Masoud *et al.* 2015). TiO_2_ NMs have also been described as mediating inhibition of the Wnt signalling pathway (Hong *et al.* 2017). | |
| **🡪KE Inflammation** | |
| **AOP173** [*Substance interaction with the lung resident cell membrane components leading to lung fibrosis*] | |
| **AOP282** [*Adverse outcome pathway on photochemical toxicity initiated by light exposure*]. | |
| **🡪KE Genotoxicity or DNA damage** | |
| AOP451 [*Interaction with lung resident cell membrane components leads to lung cancer*] | Starts with the same MIE and the same two first KEs as AOP173. AOP451 leads to the AO Lung cancer via the KEs increase, cytotoxicity; increase, reactive oxygen species; secondary genotoxicity; increased DNA damage and mutations; increase, cell proliferation, epithelial cells (Nymark *et al.* 2021). |

Table S4. A structured overview of NMs Intrinsic Hazard KPIs in σ-human health safety dimension.

| **Intrinsic Inhalation Hazard (**end-points assessment)**:** A composite KPI aimed at assessing the overall intrinsic inhalation hazard of NMs.  **-Measurement method:** Laboratory analysis including Alamar blue, LDH, DCFH_2_-DA, γ-H_2_AX assays and Annexin V/PI cell cycle analysis (cytofluorimetric) along with propidium iodide and ELISA. **Unit of measurement:** Normalization or scaling required to harmonize and merge KPIs containing numeric values (against benchmarks, references, or positive/negative controls), mass per volume values (pgr/mL), or percentage (%). **Ranges**: Various ranges per KPI defined from end-points measurements **Thresholds/target values:** Determined through expert judgment to ensure comprehensive safety evaluation.  **-Objective:** KPI minimization (KP2-5, KPI1 results of cell viability need to be inversed). | | |
| --- | --- | --- |
| KPIs. | **KPI 1:** Cell viability (%): assesses cytotoxicity using Alamar blue.  **KPI 2:** Oxidative stress (fold-change): DCFH_2_-DA assay to evaluate NMs oxidative stress induction.  **KPI 3:** DNA damage (fold-change): γ-H_2_AX assay after immunostaining with Phospho-Histone H2A.X to assess the potential of NMs to cause DNA damage.  **KPI 4:** Inflammatory potential (pg/mL or fold change): ELISA assay is employed to measure markers such as IL-8, indicating the potential inflammatory response.  **KPI 5:** Cell-NMs bio-interaction (side scatter, SSC values). SSC values obtained by cytofluorimetric analyses used as a proxy for cell and NM interaction and uptake. | |
| KDFs | **KDF 1:** Exposure conditions (i.e., concentration in µg/mL and the duration of exposure in hours). These parameters can be varied systematically in experiments to understand their impact on KPIs. | |
| PCFs | PCF 1: The intrinsic pchem properties of NMs influencing their intrinsic hazard (same as ***Table S1***).  PCF 2: The extrinsic properties of NMs influencing their behaviour in culture media and thus, their potential hazard (ECHA 2022). The properties depend on the dispersing cell medium (Dulbecco’s Modified Eagle Medium, DMEM, with 1% fetal bovine serum, FBS, pH=7.2-7.4).  Moreover, size distribution can change during time, e.g., at time 0 (t_0_) after 24 hours of exposure (t_24_) (Maiorano *et al.* 2010). To account for property alterations over time, measurements of hydrodynamic size and PdIs were performed at t_0_ and t_24._ | |
| **Intrinsic Ingestion Hazard** (end-points assessment)**:** A composite KPI aimed at assessing the overall intrinsic intestinal hazard of NMs.  **-Measurement method:** Laboratory analysis using various assays including WST-1, DHR123, and 53BP1 assay. **Unit of measurement:** Normalization or scaling required to merge KPIs. **Ranges**: Various ranges per subsequent KPI defined from measurements. **Thresholds/target values:** Determined through expert judgment.  **-Objective:** KPI minimization (KP2-3, KPI1 results of cell viability need to be inversed). | | |
| KPIs | **KPI 1:** Cell viability (%), assess cytotoxicity using assays such as WST-1.  **KPI 2:** Oxidative stress (fold-change): Evaluate NM reactivity using DHR123 assays.  **KPI 3:** DNA damage (tail % or fold-change): Assess DNA damaging potential using 53BP1 assay. | |
| KDFs | **KDF 1:** Exposure conditions (includes parameters such as the concentration in µg/mL or duration of exposure in hours and pre-treatment of NMs (e.g., digestion process that mimics ingestion). | |
| PCFs | PCFs same as above. | |
| Since TiO₂ NMs are intended for use in filters, their toxicity was also tested under conditions that mimic the storage and use phases. To achieve this, the particles were aged for 24 hours or 139 hours in a Q-SUN Xe-1 climatic chamber, set to an irradiance of 1.44 W/m² and a temperature of 40°C, and re-tested. | | |
| **Intrinsic Skin Hazard** (end-points assessment)**:** A composite indicator aimed at assessing the overall intrinsic Skin hazard of NMs **-Measurement method:** Laboratory analysis using various assays including WST-1, CellROX amd DHR123, and micronucleus and 53BP1 assay on keratinocytes.  **Unit of measurement:** Normalization or scaling required to merge KPIs. **Ranges**: Various ranges per subsequent KPI defined from measurements. **Thresholds/target values:** Determined through expert judgment.  **-Objective:** KPI minimization (KP2-3, KPI1 results of cell viability need to be inversed). | | |
| KPIs | | **KPI 1:** Cell viability (%), assesses cytotoxicity using WST-1.  **KPI 2:** Oxidative stress (fold-change): cellular tests that assess NM reactivity: CellROX & DHR123.  **KPI 3:** DNA damage (tail % or fold-change): Assess DNA damaging potential using 53BP1 and micronucleus assay. |
| KDFs | | **KDF 1:** Exposure conditions (i.e., concentration in µg/mL and the duration of exposure in hours). These parameters can be varied systematically in experiments to understand their impact on KPIs. |
| PCFs | | PCFs same as above. |
| **Intrinsic Acellular Hazard** (end-points assessment): A composite indicator aimed at assessing the overall intrinsic hazard of NMs by evaluating oxidative stress through the detection of a set of reactive oxygen species (ROS) indicators in acellular conditions.  **-Measurement method:** Laboratory analysis using various assays, including the GSH, RNO and Cys as oxidation models. **Unit of measurement**: expressed as the % of the model consumed per mg of NM. **Ranges**: 0-100%. **Thresholds/target values:** Determined based on expert judgment.  **-Objective:** KPI minimization. | | |
| KPIs | **KPI 1:** RNO oxidation to measure the capacity to decompose •OH radicals mediated photooxidation using the RNO model.  **KPI 2:** GSH oxidation: acellular test assesses oxidative potential against cell natural antioxidant defences using the thiol group assay (Ellman reagent).  **KPI 3:** Cys oxidation: acellular test assesses oxidative potential against cell natural antioxidant defences using the thiol group assay (Ellman reagent). | |
| KDFs | **KDF 1:** modification agent (uncoated/non-doped, N-doped, SiO₂-coated, N-doped and SiO₂-coated **KDF 2:** exposure dose (mg/L) and exposure duration (minutes), which influence the extent of interaction between the NMs and the models.  **KDF 3:** irradiation source (types include dark conditions and UV-light irradiation.) critical for activating the photocatalytic properties | |
| PCFs | PCF 1: The intrinsic pchem properties of NMs influencing their intrinsic hazard (same as ***Table S1***). | |

### γ-cost dimension.

Table S5. A structured overview of NMs synthesis cost effectiveness KPI of γ-cost dimension in LCS-1.

| **NMs synthesis cost effectiveness:** A composite KPI that captures synthesis cost changes as a function of KDFs to satisfy economic requirements at laboratory scale.  **-Measurement method:** LCC based on ISO 15686-5 methodology with representative costs associated to capital goods and energy, use per NM unit quantity (batch), measured in kg. The determined cost for the synthesis of the technical unit associated to 5 g of N-TiO_2_ NPs was obtained and the analysis of the major cost contributors has been carried out.  **Unit of measurement**: monetary terms. **Ranges**: Depending on the minimum and maximum values in the DoE. **Thresholds/target values:** Data restricted to project partners.  **-Objective:** KPI minimization. | |
| --- | --- |
| KPIs | **KPI 1:** Energy consumption: the amount of electricity consumed during the synthesis process (kW).  **KPI 2:** Raw materials cost used in the synthesis process: total expenditure on final reagents used in the synthesis process, expressed in euros per unit of reagent. |
| KDFs | **KDF 1**: titanium tetraisopropoxide (amount and associated costs)  **KDF 2**: ammonium hydroxide (amount and associated costs)  **KDF 4**: demineralised water (amount and associated costs)  **KDF 5**: ice (amount and associated costs)  **KDF 6:** modification agent (uncoated/non-doped, N-doped, SiO₂-coated, N-doped and SiO₂-coated. In case of tier 2 NMs the economic requirements are determined for the preparation of 1 kg of TiO_2_-@SiO_2_ NPs with main KDFs, the TiO_2_ and SiO_2_ quantities. |

## LCS-2: NMs incorporation phase

### φ-functionality dimension

Table S6. A structured overview of NEPs Functionality KPI of φ-functionality dimension in LCS-2.

| **(Textile and PMMA)** **- Functionality KPI:** A simple KPI reflecting the amount of material deposited.  **-Measurement method:** Elemental analysis of coated substrates by inductively coupled plasma - optical emission spectrometry (ICP-OES), mineralised by acidic microwave digestion. **Unit of measurement:** mg (Ti)/g (substrates). **Thresholds/target values:** The thresholds are related to the results of the photocatalytic properties assessed at LCS-3: minimum amount required to achieve a photocatalytic reduction of NOx> 90%.  **-Objective**: KPI minimisation | |
| --- | --- |
| KDFs | **KDF 1**: Suspension flow rate (mL/min). The rate at which the suspension is sprayed onto the substrate.  **KDF 2**: Number of nozzles. The quantity of nozzles used during the deposition process.  **KDF** **3**: Suspension concentration (%). NM in suspension sprayed to the substrate.  **KDF** **4**: Substrate matrix. The material composition of the fabric substrate which can affect the adhesion of NMs.  **KDF 5:** Spraying time (min). Duration of the spray application.  **KDF 6:** Plasma pre-treatment, in case of PMMA of panels. |

### σ-human health safety dimension

Table S7. A structured overview of various σ-human health safety KPIs in the LCS-2.

| **Occupational Exposure** (end-points assessment for emission study, mid-point assessment through safety assessment on human health): A composite KPI that provides estimates of worker inhalation exposure levels targeting the 95^th^ percentile of the lognormal distribution of 8-hour exposure to TiO_2_ NMs.  **-Measurement method:** A tiered exposure assessment incorporating monitoring campaigns, laboratory analysis of air particles, and probabilistic modelling. The assessment involves field techniques such as Near-Field (NF) and Far-Field (FF) campaigns, instrumentation including Scanning Mobility Particle Sizer (SMPS) and Optical Particle Counter (OPC) (Del Secco *et al.* 2022, Belosi *et al.* 2023). Probabilistic modelling is employed for estimating workers’ exposure levels and safety (Koivisto *et al.* 2021, Koivisto *et al.* 2022b). The regulated strategy for evaluating exposure to agents by inhalation according to EN 689 was also used, by determining the concentration of TiO_2_NMs in the worker’s breathing zone. The samples of respirable fraction were captured using a cyclone and a mixed cellulose membrane filter. The concentration of Ti in the filters was determined with inductively coupled plasma mass spectrometry (ICP-MS). **Unit of measurement:** Ti-μg/m^3^ **Ranges:** Defined by the instruments. **Thresholds/target values:** Comparison with Occupational Exposure Limit (OEL), in our case the NIOSH Recommended Exposure Limit (REL) values given as respirable fraction is 300 μg/m^3^ for ultrafine TiO_2_ as 8-hour total weight average (TWA). CoU is considered adequate when the 95th percentile of the lognormal distribution of 8-hour exposure is below 0.1×REL.  **-Objective:** KPIs minimization. | |
| --- | --- |
| KPIs. | **KPI 1**: Emission concentrations (expressed as mass μg/m^3^, particle number and surface concentration μm^2^/cm^-3^) measured in various settings including the background, spray chamber, NF and FF locations using SMPS and OPC and off-line gravimetric analysis of particles (effective densities, ICP-MS).  **KPI 2**: Emission rates (mg/min) determined by a mass flow model, that reproduce the test specific mass concentrations in NF.  **KPI 3**: Simulated exposure levels under diverse OCs utilizing a probabilistic exposure model. The assessment is based on RWC scenarios, ensuring a conservative estimation of exposure levels. |
| KDFs | **KDF 1**: Suspension flow rate (mL/min). The rate at which the suspension is sprayed onto the substrate.  **KDF 2**: Number of nozzles. The quantity of nozzles used during the deposition process.  **KDF** **3**: Suspension concentration (%). NM in suspension sprayed to the substrate.  **KDF** **4**: Substrate matrix. The material composition of the substrate which can affect the adhesion of NMs.  **KDF 5:** Spraying time (min). Duration of the spray application.  **KDF 5**: LEV ventilation ratio (particularly the NF/FF air exchange rates (m^3^/min)) |
| **Inhalation risk assessment** (emissions: end-point, modelling: mid-point)**:** A composite KPI for the realistic occupational inhalation hazard.  **-Measurement method:** A multi-tier NAM approach integrating emission campaigns, MPPD modelling, dose translation, and *in vitro* testing (Motta *et al.* 2024). The alveolar retained doses are calculated with MPPD 4.0 and translated in real occupational monthly or yearly human exposure doses then tested in an *in vitro* co-culture model representative of the alveolar space (A549 + THP1-derived macrophages cells). Exposures are performed at the air liquid interface, to mimic actual inhalation and deposition. **Unit of measurement**: % **Ranges**: 1-100% **Thresholds/target values:** through expert interpretation and the usage of controls during the *in vitro* testing.  **-Objective:** KPI1, 2, 4 minimizations. KPI3 maximization. | |
| KPIs with KDFs and PCFs | **KPI 1**: Emission concentrations (μg/m^3^) measured in NF from emission campaigns representing the inhalation external dose. |
|  | **KPI 2:** Deposited internal lung dose by MPPD modelling (alveolar deposited fraction, μg/cm^2^)  PCF 1: nanoparticle primary size (nm)  PCF 2: effective densities (g/cm^3^). Reflects the mass per unit volume of NMs, influencing their dispersion and fate in biological systems (Trabucco *et al.* 2022).  PCF 3: dissolution rate (%) in simulated lung fluids (1) lung lining fluids 2) phagolysosomal simulated fluid in lung alveolar macrophages after phagocytosis (uptake) of NM to infer biodurability and bioaccessibility of NMs using in vitro acellular dissolution (ISO/TR19057 guideline (ISO 2017) and analysed by ICP-MS to determine the ionic concentration in the soluble fractions. The GRACIOUS inhalation IATA was used to rank the NMs dissolution profiles^[[1]](#footnote-1)^.  PCF 4: hydrodynamic diameter by DLS, in simulated lung fluids at time points 0h, 24h and 48h  PCF 5: zeta potential by DLS using a Zetasizer NanoSeries ZS90 (Malvern Instruments), in simulated lung fluids at time points 0h, 24h and 48h |
|  | **KPI 3:** Deposition efficiency of NMs in a system (e.g., Vitrocell® Cloud α 12 equipped with a nebulizer or RFS – Cultex Compact module to achieve a desired concentration (ng/cm^2^). |
|  | **KPI 4:** Hazard response: Evaluates the toxicological responses, focusing on *in vitro* inflammation end-points and cytotoxicity markers (IL-8, IL-6, and IL-1β and LDH). Tier 2 advanced *in vitro* models, co-cultures A549 and alveolar macrophages derived from differentiated THP-1 monocytes.  **KDF 1**: Exposure conditions (time of exposure i.e 1,6 and 12 months and dose of exposure calculated and used for *in vitro* studies (µg/cm^2^)  PCF 1: The intrinsic pchem properties of NMs (same as ***Table S1***).  PCF 2: The extrinsic pchem properties of NMs in a co-culture model of alveolar space. |
| **Population Inhalation Exposure-Burden of Disease** (mid-point assessment)**:** A KPI that assesses the general population the safety of the general population expressed as Disability Adjusted Life Years (DALYs).  **-Measurement method:** A multi-tier air emission assessment based on emission monitoring campaigns and a bi-Gaussian plume model IMPACT (Immission Prognosis Air Concentration Tool) to calculate the industrial, residential, traffic, and agricultural emissions impact on the air concentrations and depositions on a local scale (grid set as 2 × 2 km) under RWC conditions assuming highest production volumes and material usage in full capacity production where machine is assumed operates 5d/w through a year and 8h/d (Koivisto *et al.* 2022a, Koivisto *et al.* 2022b) Utilizes the FF monitoring campaigns to model emission factors and rates (mg-Ti/min). **Unit of measurement**: Absolute metric, **Ranges**: Defined by modelling simulations. **Thresholds/target values:** The acceptable risk range is typically defined as one additional cancer in 1,000,000 persons per year e.g., by WHO for risk characterization of ambient air pollution.  **-Objective:** KPI minimization. | |
| KPI with KDFs | **KPI 1**: FF emission concentrations (μg/m^3^) calculated for RWC conditions  **KPI 2**: Process emissions factors (from room to local exhaust ventilation (LEV) which results into modelling NMs released to the outdoor air, i.e., fugitive emissions via LEV exhaust).  **KPI 3**: Transfer efficiency (%). A fraction that is deposited to the substrate during spraying  **KDF 1**: Suspension flow rate (mL/min). The rate at which the suspension is sprayed onto the substrate.  **KDF 2**: Number of nozzles. The quantity of nozzles used during the deposition process.  **KDF** **3**: Suspension concentration (%). NM in suspension sprayed to the fabric.  **KDF** **4**: Substrate matrix. The material composition of the fabric substrate which can affect the adhesion of NMs.  **KDF 5**: LEV efficiency (mg- NM/g -NM) as the ratio of the NM release and use and subsequent ventilation ratio (particularly the NF/FF air exchange rates (m^3^/min).  PCF 1: nanoparticle primary size (nm) |

### ε-environment dimension

Table S8. A structured overview of Environmental Exposure KPI in ε-environment dimension of LCS-2.

| **Environmental Exposure (**mid-point assessment)**:** Simulated concentrations of NMs in the soil top layer. To cover seasonal variation in meteorology, the ground level concentrations and deposition fluxes (accumulation) are given as annual averages.  **-Measurement method:** Utilizes emission monitoring campaigns (Del Secco *et al.* 2022, Belosi *et al.* 2023), a mechanistic model analysing mass flows associated to LEV filter (Koivisto *et al.* 2022b), and a single compartment model (bi-Gaussian plume model- IMPACT) estimating the accumulation concentrations of NMs in the soil top layer (Koivisto *et al.* 2022a) according to ECHA Chapter R.16, A.16-3.3.6 “Calculation of PEC_local_ for the soil compartment”. **Unit of measurement**: ng-Ti/m^3^. **Ranges**: Defined by modelling simulations such as weather conditions and source parameters (source location and height, source type, etc.,). Values are compared with thresholds levels in a 10-year production scenario. **Thresholds/target values:** Limit values of 0.1 μg-Ti/m^3^ derived from OEL (lower range) for nanosized Ti. Values are compared with thresholds levels in a 10-year production scenario. These limit values are indicative framework values used in this study.  **-Objective:** KPI minimization. | |
| --- | --- |
|  | **KPI 1**: Emission concentrations (μg/m^3^) calculated for RWC conditions  **KPI 2**: Process emissions factors (mg of NMs per g of sprayed NMs) ratio of NM release and use which results into modelling NMs emissions to outdoor air via LEV exhaust (mg-NM/min)  **KPI 3**: Transfer efficiency (%). A fraction that is deposited to the substrate during spraying |
| KDFs | **KDF 1**: Suspension flow rate (mL/min). The rate at which the suspension is sprayed onto the substrate. Specific flow rates correspond to a specific number of nozzles.  **KDF 2**: Number of nozzles. The quantity of nozzles used during the deposition process.  **KDF** **3**: Suspension concentration (%). NM in suspension sprayed to the substrate.  **KDF** **4**: Substrate matrix. The material composition of the substrate which can affect the adhesion of NMs.  **KDF 5**: LEV efficiency (mg-NP/g-NP). |

### γ-cost dimension.

Table S9. A structured overview of NMs incorporation cost effectiveness in γ-cost dimensions of LCS-2.

| **NMs incorporation cost effectiveness:** A composite KPI capturing the cost changes as a function of KDFs.  **-Measurement method:** LCC methods specific to economic evaluation in industrial processes according to ISO 15686. Representative costs in the analysis are the ones associated to: reagents, energy, and infrastructure use per NEP unit quantity. An example is the unit cost for treating 1 m2 of textile surface.  **Unit of measurement**: The cost ratio is calculated by normalizing the specific cost by the amount of TiO_2_ -N NMs deposited and dividing it by the maximum normalized cost across all tests. **Ranges**: Depend on the spray coating machinery. **Thresholds/target values:** the time and % of cost ratio referred to the maximum specific cost to reach a degradation of the pollutant equal to 50% of its initial concentration.  **-Objective:** KPI minimization. | |
| --- | --- |
| KPIs with KDFs | **KPI 1:** Energy Consumption: Measures the amount of electricity consumed during the manufacturing process (kW). Sum of energy consumption of all the steps and auxiliaries.  **KDF 1**: Suspension flow rate (mL/min). The rate at which the suspension is sprayed onto the substrate.  **KDF 2**: Number of nozzles. The quantity of nozzles used during the deposition process.  **KDF 3**: Quantity of TiO_2_NMs deposited on the substrateper unit mass (μg/g) or surface area (μg/cm²), determined by ICP-MS  **KDF 4**: Conveyor belt speed (i.e. m/min)  **KDF 5:** irradiation source (types include visible light and UV-light), and time (in minutes) critical for activating the photocatalytic properties and for the energy consumption,  **KDF 3:** Plasma pre-treatment, in case of PMMA panels.  **KDF 4:** Thermal pre-treatment temperature (°C) , in case of textile substrates.  **KDF** **5**: Substrate matrix. The material composition of the substrate which can affect the adhesion of NMs. |

## LCS-3: NEPs use phase

### φ-functionality dimension

Table S10. A structured overview of NEPs Functionality KPI in φ-functionality dimension of LCS-3.

| **Textile Functionality:** A composite KPI reflecting the photocatalytic efficiency of NEPs and the technical quality and durability of NEPs.  **-Measurement method****:** Laboratory analysis conducted according to photodegradation tests by reducing Nitrogen Oxide and/or nitrogen dioxide (NOx) gas using a chemiluminescence for the evaluation of abatement of gaseous pollutants under visible light activation (internal protocol, Colorobbia).  Laboratory analysis according to an adaptation of the ISO 105 X12:2016 - Part X12: Colour fastness to rubbing for abrasion resistance, and EN ISO 105-C06 A1S for washing stability.  The percentage of Ti amount remaining on the substrate determines the functionality, evaluated by ICP-MS  **Unit of measurement:** expressed as reduction %. **Ranges**: 0-100%. **Thresholds/target values:** The NEPs undergoes quality and stability tests if photocatalytic efficiency is > 90%.  -**-Objective:** KPI maximization. | |
| --- | --- |
| KPIs | **KPI 1**: Photocatalytic performance of NEPs.  **KPI 2**: Washing stability: Reflects the retained functionality of the NEPs after various washing cycles.  **KPI** **3**: Abrasion resistance: Measures the ability of the NEPs (Ti unit mass (μg/g) amount deposited, per area of substrate (cm^2^) confirmed by particle distribution using ICP-MS) to withstand mechanical abrasion without breakdown.  **KPI 4**: visual assessment of substrate colourability |
| KDFs | **KDF 1**: Quantity of Ti deposited and remaining on the substrate per unit mass (μg/g) or surface area (cm²), determined by ICP-MS  **KDF 2**: Number of abrasion cycles. Adjusting these factors helps tailor the product for specific use-case scenarios, potentially reducing the release of NMs.  **KDF 3**: Washing cycles. The number and conditions of washing cycles. |

### σ-human health safety dimension

Table S11. A structured overview of NEPs Consumers exposure KPI in φ- human health safety dimension of LCS-3.

| **-Consumers Exposure (**end-point assessment)**:** A composite KPI that assesses inhalation exposure under RWC.  **-Measurement method:** KPI based on exposure assessment including monitoring campaigns by means of an external Condensation Particle Counter (CPC, TSI mod. 3775) and a low-cost optical particle counter Alphasense (OPC-N3) to determine possible release emissions and lab analysis of air particles (iCAP RQ ICP-MS) and Modelling by a deterministic single compartment model (Task Exposure Assessment Simulator (TEAS)). The KPI provides consumers exposure estimates 95th percentile 8-hour exposure (μg Ti /m3) which is compared to reference values to ensure consumers safety. TiO_2_ release was studied under laboratory-simulated conditions. The TiO_2_ particle release factor was measured in scalable units according to the photoactive surface area and volume flow (ng TiO_2_/m2×m3). The impact of Gearbox Wivactive on indoor concentration level under reasonable worst-case conditions was predicted by using the release factor and a well-mixed indoor aerosol model.  **Unit of measurement**: μg Ti /m3. **Ranges**: Defined by modelling simulations **Thresholds/target values:** Current proposed OELs vary for nano-TiO_2_ from 0.8 to 5000 μg/m^3^ when given in different size fractions and specified under different experimental conditions for 8-hour time weighted average  **-Objective:** KPI minimization. | |
| --- | --- |
| KPIs with KDFs | **KPI 1**: Emission concentrations (ng/ m^2^ of photoactive surface area × m^3^ of ventilated air) inside the box, outside the box. Instruments: CPC & OPC-N3.  **KPI 2**: Emission flow rates, ng/h. The flow rate was determined by averaging the inlet velocities measured by means of a hot wire anemometer (Terman ANM-0/B, LSI spa, Milan, Italy).  **KPI 3**: particle release factor was measured in scalable units according to the photoactive surface area and volume flow (ng TiO_2_/m2×m3).  **KPI 4**: Simulated exposure levels under realistic conditions using a single compartment mass flow model (Task Exposure Assessment Simulator (TEAS) 2019 V 1.00, model No. 101, Exposure Assessment Solutions, Inc., Morgantown, MI, USA). |
|  | **KDF 1:** Ventilation system (in our case Quartz-Microfibers filter- Grade T293, Sartorius) and chamber ventilation air volume flow rate to estimate the emission rate from the OPC-N3 concentration time series.  Fully mixed concentrations and no particle losses inside the chamber were assumed. |

### ε-environment dimension

Table S12. A structured overview of Environment Exposure KPI in ε-environment dimension of LCS-3.

| **Textiles** **Release KPI** (end-point assessment): A KPI showcasing quantification of NMs released into washing waters accompanied by biological fate uptake kinetics parameters focusing on dissolution in environmentally relevant media and bioaccumulation in soil invertebrates.  **-Measurement method:** An approach containing quantification of NMs released into washing waters using EN ISO 105-C06 (A1S) and ICP-MS. Environmental fate parameters are collected; bioaccumulation assessment for soils to obtain kinetic parameters (OECD 317). *Enchytraeus crypticus* are introduced for a 14 days elimination phase (this is shorter than the 21 days recommended by the TG, but was deemed sufficient based on expert judgement). Ti concentration in organisms measured by ICP-MS. **Unit of measurement**: expressed in terms of total particles concentration and dissolved fraction) of NMs in washing water after washing simulations. **Ranges**: None. **Thresholds/target values:** Determined through expert judgment.  **-Objective:** KPI minimization. | |
| --- | --- |
| KDF | **KDF** **1**: Number of washing cycles. The number and conditions of washing cycles.  **KDF 2**: Quantity of NMs deposited on the substrate per unit mass (μg/g), determined by ICP-MS. |
| PCFs | PCF 1: Bioaccumulation rate using *Enchytraeus crypticus*.  Alongside pchem are environmental factors, such as pH or organic matter content, that influence NM fate and behaviour, and ultimately exposure concentrations. |

## LCS-4: NEPs EoL

### ε-environment dimension

Table S13. A structured overview of NEPs Consumers exposure KPI in φ- human health safety dimension of LCS-3.

| **Honeycombs (Ceramic and Polycarbonate) NEPs - Thermal treatment KPI:** A KPI that shows the release during the efficiency recovery of NEPs during a thermal treatment  **-Measurement method:** Release studies were performed during the heating process by an in-situ air monitoring campaign using SMPS and OPS, and then, TEM coupled to EDS was used to characterize the particles released in the air (Internal protocol by Colorobbia). **Unit of measurement:** particle concentration (particles/m^3^). **Thresholds/target values:** Defined by laboratory instruments.  **-Objective**: KPI minimisation | |
| --- | --- |
| KDFs | **KDF 1**: Quantity of Ti initially deposited and remaining on the substrate per unit mass (μg/g) or per surface area (μg/cm²), determined by ICP-MS  **KDF 2:** Usage conditions (duration and frequency). |
| **Polycarbonate honeycombs NEPs regeneration - Mild soap cleaning KPI:** A KPI that shows the release during the efficiency recovery of NEPs during a thermal treatment  **-Measurement method:** A washing methodology with distilled water and mild soap was followed (Internal protocol by Colorobbia). Ti release into washing waters and rinsing waters were determined by ICP-MS.  **Unit of measurement:** Ti released (%). **Thresholds/target values:** Defined by laboratory instruments.  **-Objective**: KPI minimisation | |
| KDFs | **KDF 1**: Quantity of Ti initially deposited per surface area (cm²), determined by ICP-MS  **KDF 2:** Usage conditions (duration and frequency). |
| **Honeycombs (Ceramic and Polycarbonate) NEPs - Landfill simulation KPI:** A KPI that shows the release during landfilling conditions.  **-Measurement method:** Leaching experiments based on soft abrasion test (standardized protocol EPA Standard Method 1311: “Toxicity Characteristic Leaching Procedure (1992)” to simulate the end-of-life stage under landfilling conditions. A qualitative solid-liquid separation was done by cellulose filter and aliquots of the leachate were taken to ICP-MS, mineralised by acidic microwave digestion and TEM.  **Unit of measurement:** μg Ti/g sample (Ti mass release) or Ti release (%). **Thresholds/target values:** Defined by laboratory instruments.  **-Objective**: KPI minimisation | |
| KDFs | **KDF 1**: Quantity of Ti initially deposited per surface area (cm²), determined by ICP-MS  **KDF 2:** Usage conditions (duration and frequency). |

# References

Belosi, F., et al. (2023). "Critical aspects in occupational exposure assessment with different aerosol metrics in an industrial spray coating process." NanoImpact **30**: 100459.

Braakhuis, H. M., et al. (2021). "Mechanism of Action of TiO(2): Recommendations to Reduce Uncertainties Related to Carcinogenic Potential." Annu Rev Pharmacol Toxicol **61**: 203-223.

Brand, W., et al. (2020). "Possible effects of titanium dioxide particles on human liver, intestinal tissue, spleen and kidney after oral exposure." Nanotoxicology **14**(7): 985-1007.

Del Secco, B., et al. (2022). "Particles Emission from an Industrial Spray Coating Process Using Nano-Materials." Nanomaterials **12**(3): 313.

ECHA (2022). "Appendix for nanoforms applicable to the Guidance on Registration and Substance Identification."

Gerloff, K., et al. (2017). "The Adverse Outcome Pathway approach in nanotoxicology." Computational Toxicology **1**: 3-11.

Hong, F., et al. (2017). "Nanoparticulate TiO(2) -mediated inhibition of the Wnt signaling pathway causes dendritic development disorder in cultured rat hippocampal neurons." J Biomed Mater Res A **105**(8): 2139-2149.

Koivisto, A., et al. (2021). "Assessment of exposure determinants and exposure levels by using stationary concentration measurements and a probabilistic near-field/far-field exposure model [version 1; peer review: 2 approved]." Open Research Europe **1**(72).

Koivisto, A. J., et al. (2022a). "Burden of Disease (BoD) Assessment to Estimate Risk Factors Impact in a Real Nanomanufacturing Scenario." Nanomaterials **12**(22): 4089.

Koivisto, A. J., et al. (2022b). "Quantifying Emission Factors and Setting Conditions of Use According to ECHA Chapter R.14 for a Spray Process Designed for Nanocoatings-A Case Study." Nanomaterials (Basel) **12**(4).

Maiorano, G., et al. (2010). "Effects of cell culture media on the dynamic formation of protein-nanoparticle complexes and influence on the cellular response." ACS Nano **4**(12): 7481-7491.

Masoud, R., et al. (2015). "Titanium Dioxide Nanoparticles Increase Superoxide Anion Production by Acting on NADPH Oxidase." PLOS ONE **10**(12): e0144829.

Motta, G., et al. (2024). "An integrated new approach methodology for inhalation risk assessment of Safe and Sustainable by Design nanomaterials." Environment International: 108420.

Nymark, P., et al. (2021). "Adverse Outcome Pathway Development for Assessment of Lung Carcinogenicity by Nanoparticles." **3**.

OECD (2023). Test No. 125: Nanomaterial Particle Size and Size Distribution of Nanomaterials.

Trabucco, S., et al. (2022). "Measuring TiO2N and AgHEC Airborne Particle Density during a Spray Coating Process." Toxics **10**(9): 498.

1. instantaneously dissolving, when NMs half-life time (T½) is < 10 min in LLF; quickly dissolving, T½ < 48 h in LLF or PSF; gradually dissolving, T½ > 48 h but < 60 days in LLF or PSF; or very slowly dissolving, T½ > 60 days in PSF. [↑](#footnote-ref-1)
